# Supplementary material for: Pixel super-resolution with spatially entangled photons
Source: Nat Commun. 2022 Jun 22;13:3566. doi: 10.1038/s41467-022-31052-6 (PMC9217946; doi:10.1038/s41467-022-31052-6)
Supplement: Supplementary file 1 — Supplementary Information [file 41467_2022_31052_MOESM1_ESM.pdf]

Supplementary information: Pixel super-resolution with spatially-entangled photons

Hugo Defienne[1,*], Patrick Cameron[1], Bienvenu Ndagano[1], Ashley Lyons[1], Matthew Reichert[2], Jiuxuan Zhao[3], Andrew R. Harvey[1], Edoardo Charbon[3], Jason W. Fleischer[2], Daniele Faccio[1,†]

[1]*School of Physics and Astronomy, University of Glasgow, Glasgow G12 8QQ, UK*

[2]*Department of electrical and computer engineering, Princeton University, Princeton, USA*

[3]*Advanced Quantum Architecture Laboratory (AQUA), Ecole Polytechnique Federale de Lausanne (EPFL), 2002 Neuchatel, Switzerland*

1 Details on the joint probability distribution (JPD) measurement

This section provides more details about the measurement of the spatially resolved JPD of photon pairs using a Electron Multiplied Charge Coupled Device (EMCCD) or single-photon avalanche diode (SPAD) camera. Further theoretical details can be found in [1], [2] and [3].

1.1 JPD measurement.

EMCCD ans SPAD cameras can be used to reconstruct discrete (a) intensity distribution I_{mn} and (b) spatial joint probability distribution Γ_{ijkl} of photon pairs, where (m, n), (i, j) and (k, l) are pixel labels corresponding to spatial positions (x_m, y_n), (x_i, y_j) and (x_k, y_l), respectively. To do that, the camera first acquires a set of $M + 1$ frames $\{I^{(l)}\}_{l \in [\![1, M+1]\!]}$ using a fixed exposure time and without using a threshold in the case of EMCCD camera. Then:

(a) The intensity image is reconstructed by averaging over all the frames:

$$I_{mn} = \frac{1}{M} \sum_{l=1}^{M} I_{mn}^{(l)} \tag{1}$$

(b) The JPD is reconstructed by performing the following substation:

$$\Gamma_{ijkl} = \frac{1}{M} \sum_{l=1}^{M} I_{ij}^{(l)} I_{kl}^{(l)} - \frac{1}{M} \sum_{l=1}^{M} I_{ij}^{(l)} I_{kl}^{(l+1)} \tag{2}$$

Under illumination by the photon pairs, intensity correlations in the left term of Equation 2 originate from detections of both real coincidences (two photons from the same entangled pair) and accidental coincidences (two photons from two different entangled pairs), while intensity correlations in the second term originate only from photons from different entangled pairs (accidental coincidence) because there is zero probability for two photons from the same entangled pair to be detected in two successive images. A subtraction between these two terms leaves only genuine coincidences, that are proportional to the spatial JPD of the pairs.

1.2 Interpolation of some JPD values measured with an EMCCD camera.

Using an EMCCD camera, not all correlation values of the JPD can be measured using Equation 2, in particular those (a) at the same pixel i.e. Γ_{ijij} and (b) and those between vertical neighboring pixels i.e. $\Gamma_{iji\,(j\pm l)}$:

(a) As demonstrated in theory in [1], Equation 2 is not valid for $(i, j) = (k, l)$. For example, Figure 1.a shows a conditional image Γ_{ijkl} for a reference pixel $(i, j) = (-5, 3)$ obtained from the JPD measured in the experiment described in Figure 1.a of the manuscript with a 16 μm pixel pitch camera and using Equation 2. In this image, we observe that the value at $(k, l) = (-5, 3)$ is more than 10^3 times brighter than the other images values, confirming that this value is wrong. In practice, this issue is solved by interpolating all the values in the diagonal Γ_{ijij} using neighbouring correlation values on the same row: $[\Gamma_{ij\,(i+1)\,j} + \Gamma_{ij\,(i-1)\,j}]/2 \rightarrow \Gamma_{ijij}$. Figure 1.b shows the conditional image Γ_{ijkl} for $(i, j) = (-5, 3)$ obtained after interpolating the pixel value at $(k, l) = (-5, 3)$. More details in [2].

(b) However, we still observe in Figure 1.b the presence of artefacts in the values at positions $(k, l) = (-5, 3 \pm l)$, where l is an integer that defines the position of a pixel above or bellow $(-5, 3)$. These artefacts are due to cross-talk between pixels located on the same column of the EMCCD camera, originating from the so-called charge smearing effect [2]. In practice, these values are interpolated using neighboring correlation values on the same row: $[\Gamma_{ij\,(i+1)\,(j\pm l)} + \Gamma_{ij\,(i-1)\,(j\pm l)}]/2 \rightarrow \Gamma_{iji\,(j\pm l)}$. Figure 1.c shows the resulting conditional image Γ_{ijkl} for $(i, j) = (-5, 3)$ after interpolating the pixel values.

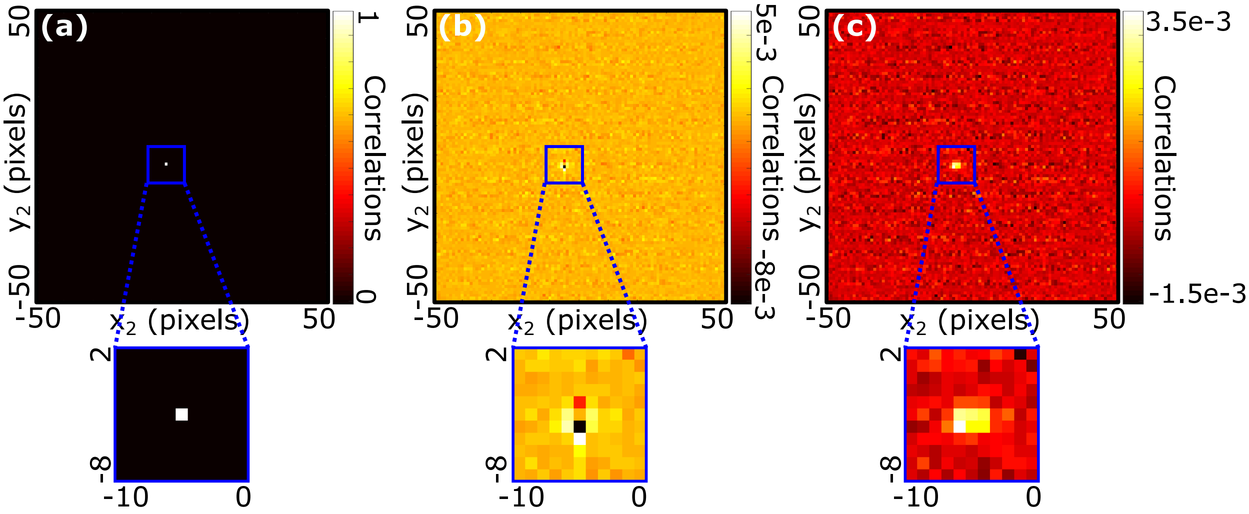

Supplementary Figure 1: ***Interpolation of JPD values when using EMCCD camera.*** ***(a)*** *Conditional image Γ_{ijkl} for a reference pixel $(i, j) = (-5, 3)$ directly obtained from the JPD measured in the experiment described in Figure 1.a without interpolating the pixel values.* ***(b)*** *Same conditional image obtained after interpolating the diagonal values Γ_{ijij} of the measured JPD.* ***(c)*** *Same conditional image obtained after interpolating all the values $\Gamma_{iji\,(j\pm q)}$ of the measured JPD, where q is an integer that correspond to an arbitrary number of vertical pixel above or bellow $\mathbf{r}$, and $\mathbf{e_y}$ is the unit vector pixel in the y-axis.*

1.3 Residual term in the measured JPD using EMCCD camera

The use of Equation 2 to measure the JPD with an EMCCD camera is not perfect and a residual background $\mathcal{B}$ is also retrieved: $\Gamma_{ijkl} + \mathcal{B}$. This problem is studied in [1]. This background term originates from the fluctuations of the EMCCD camera gain during the acquisition process, for example due to temperature drifts and variations in the high voltage clock amplitude. $\mathcal{B}$ is a complex term proportional to $\alpha I_{ij} I_{kl} + \beta(I_{ij} + I_{kl}) + \gamma$, where I_{ij} is the intensity value at pixel (i, j), and α, β and γ are constant experimental parameters depending on the mean and variance values of

the camera gain and electronic noise. In practice, $\mathcal{B}$ is often negligible compared to Γ and can be ignored in most experiments. However, because Γ and $\mathcal{B}$ do not have the same spatial frequency content, spatial filtering effects can sometimes create asymmetric losses and level these two terms in favor of $\mathcal{B}$.

1.4 Correspondence between continuous and discrete JPD

In the experiments shown in our work, the measured JPD Γ takes the form of a 4-dimensional matrix containing $(N_Y \times N_X)^4$ elements, where $N_Y \times N_X$ corresponds the size of the illuminated region of the camera sensor. An element of the matrix is written Γ_{ijkl}, where (i, j) and (k, l) are pixel labels corresponding to spatial positions (x_i, y_j) and (x_k, y_l). Γ_{ijkl} is a discrete version of the continuous JPD $\Gamma(\mathbf{r_1}, \mathbf{r_2}) = |\Psi(\mathbf{r_1}, \mathbf{r_2})|^2$ where Ψ is the spatial two-photon wave-function associated with photon pairs, and $\mathbf{r_1} = x_1\mathbf{e_x} + y_1\mathbf{e_y}$ and $\mathbf{r_2} = x_2\mathbf{e_x} + y_2\mathbf{e_y}$ are transverse spatial positions ($\mathbf{e_x}$ and $\mathbf{e_y}$ are unit vectors along the x and y axes, respectively). Such a continous formalism is used in many theoritical works describing the propagation of spatially-entangled photon pairs [4,5]. In our work, the information content of Γ is analyzed using two types of projections:

1. In theory, the sum-coordinate projection is defined as:

$$P^+(\mathbf{r}^+) = \int \Gamma(\mathbf{r}, \mathbf{r}^+ - \mathbf{r}) d\mathbf{r}. \tag{3}$$

   where $\mathbf{r} = x\mathbf{e_x} + y\mathbf{e_y}$. It represents the probability of detecting pairs of photons generated in all symmetric directions relative to the position $\mathbf{r}^+ = x^+\mathbf{e_x} + y^+\mathbf{e_y}$. In practice, it is

calculated using the discrete space formula:

$$P^+_{i^+j^+} = \sum_{i=1}^{N_X} \sum_{j=1}^{N_Y} \Gamma_{(i^+-i)\,(j^+-j)\,i\,j}. \tag{4}$$

2. In theory, the sum-coordinate projection is defined as:

$$P^-(\mathbf{r}^-) = \int \Gamma(\mathbf{r}, \mathbf{r}^- + \mathbf{r}) d\mathbf{r}. \tag{5}$$

where $\mathbf{r} = x\mathbf{e_x} + y\mathbf{e_y}$. This represents the probability for two photons of a pair to be detected in coincidence between pairs of pixels separated by an oriented distance $\mathbf{r}^- = x^-\mathbf{e_x} + y^-\mathbf{e_y}$. In practice, it is calculated using the discrete space formula:

$$P^-_{i^-j^-} = \sum_{i=1}^{N_X} \sum_{j=1}^{N_Y} \Gamma_{(i^-+i)\,(j^-+j)\,i\,j}. \tag{6}$$

2 Theory of pixel JPD super-resolution

This section provides a theoretical description of JPD pixel super-resolution. For clarity, calculations are performed in the case of a uni-dimensional system i.e. one transverse axis x. Generalization to the bi-dimensional case is discussed at the end of the section.

2.1 Discrete JPD measurement

Figure 2.a illustrates the generic imaging configuration used in our work. Photon pairs, characterized by a spatial two-photon wave-function $\Psi_t(x_1, x_2)$ in the object plane, illuminate an unidi-

mensional object $t(x)$ that is then imaged onto a camera. Propagation of photon-pairs through the imaging system allows calculating the two-photon field in the camera plane [6]:

$$\Psi(x_2', x_1') = \iint t(x_1)t(x_2)\, \Psi_t(x_1, x_2)h(x_1' - x_1)h(x_2' - x_2)dx_1dx_2, \tag{7}$$

where Ψ is the two-photon wave-function in the camera plane, Ψ_t is the two-photon wave-function in the object plane and h is the coherent point spread function (PSF) associated with the imaging system. A camera with pixel pitch Δ and gap δ is then used to detect photon-pair correlations. It returns a discrete JPD Γ_{kl}, where k and l define two pixels centered at spatial positions x_k and x_l, respectively. A value Γ_{kl} is obtained by integrating $|\Psi(x_1, x_2)|^2$ over the corresponding pixel areas:

$$\Gamma_{kl} = \int_{x_k - \frac{\Delta-\delta}{2}}^{x_k + \frac{\Delta-\delta}{2}} \int_{x_l - \frac{\Delta-\delta}{2}}^{x_l + \frac{\Delta-\delta}{2}} |\Psi(x_1, x_2)|^2 dx_1 dx_2. \tag{8}$$

2.2 Imaging system limited by the sensor spatial resolution

A central aspect in our study is that the spatial resolution of the imaging system is limited by the camera resolution.

2.3 Experimental constraints

In practice, this condition is verified under two experimental constraints:

(c1) The highest spatial frequency component of the optical field in the object plane must be smaller

than the imaging system optical spatial frequency cut-off (i.e. not diffraction-limited).

(c2) The highest spatial frequency component of the optical field in the camera plane must be larger than the Nyquist frequency $1/(2\Delta)$, where Δ is the pixel pitch (i.e. under-sampling).

Under these constraints, the resulting image is sampled less than twice the highest frequency present in it, which creates aliasing. Note also that, assuming that photon-pairs have a correlation width σ in the camera plane (see next section), the constraint (c2) implies that $\Delta > \sigma$. In practice, observing the presence of aliasing in the measured image is a sufficient condition to ensure that both constraints (c1) and (c2) are verified.

2.3.1 Simplification of Γ_{kl}

If (c1) is verified, the imaging system PSF can be approximated as $h(x) = \delta(x)$, where δ is a Dirac-delta function (for simplicity we assumed a unity magnification), and equation 8 simplifies into:

$$\Gamma_{kl} = \int_{x_k - \frac{\Delta-\delta}{2}}^{x_k + \frac{\Delta-\delta}{2}} \int_{x_l - \frac{\Delta-\delta}{2}}^{x_l + \frac{\Delta-\delta}{2}} |t(x_1)t(x_2)|^2 |\Psi_t(x_1, x_2)|^2 dx_1 dx_2, \tag{9}$$

where t is the object and Ψ_t the two-photon wave-function in the object plane.

2.4 Model of the two-photon wave-function

The two-photon wave-function $\Psi_0(x_1, x_2)$ associated with photon pairs produced by type-I SPDC at the output of a thin non-linear crystal can be approximated using a Gaussian model [4]:

$$\Psi_0(x_1, x_2) = A \exp\left(\frac{-|x_1 - x_2|^2}{4\sigma_{r_0}^2}\right) \exp\left(\frac{-|x_1 + x_2|^2 \sigma_{\theta_0}^2}{4\lambda_p^2}\right) \tag{10}$$

where σ_{r_0} and σ_{θ_0} are the position and angular correlation width of photons at the output of the crystal, respectively, and A is a constant. Angular and position correlation widths can be written in function of the crystal thickness L, pump laser wavelength λ_p and waist ω, using the formulas:

$$\sigma_{r_0} = \sqrt{\frac{\alpha \lambda_p L}{2\pi}} \tag{11}$$

$$\sigma_{\theta_0} = \frac{\lambda_p}{2\omega}, \tag{12}$$

where $\alpha = 0.455$ [7]. It is important to note that σ_{r_0} and σ_{θ_0} are the correlation widths at the output of the crystal. Correlation width values in the camera plane - that are relevant in our imaging experiments - are calculated from the correlations in the crystal plane after taking into account the specific arrangement and magnification of the imaging system (see Tables 1 and 2).

2.4.1 Two-photon field in the object plane

Spatial shape of the two-photon field in the object plane Ψ_t depends of the experimental arrangement between the object plane and the crystal output:

Near-field illumination configuration Using a near-field imaging configuration, the output surface of the non-linear crystal is imaged onto the object. This configuration is used in the experiments shown in Figure 1 and 2 of the manuscript, and Figure 10. The two-photon field is written:

$$\Psi_t(x_1, x_2) = A' \exp\left(\frac{-|x_1 - x_2|^2}{4m^2\sigma_r^2}\right) \exp\left(\frac{-|x_1 + x_2|^2\sigma_\theta^2}{4m^2\lambda_p^2}\right) \tag{13}$$

where m is the magnification of the imaging system between the crystal output plane and the object plane, σ_r and σ_θ are position and momentum correlation width in the object plane, respectively, and A' is an amplitude parameter. In such a configuration, the position correlation width is the relevant width characterizing how strongly photon pairs are correlated when they interact with the object and imaged onto the camera.

Far-field illumination configurations Using a far-field imaging configuration, the output surface of the non-linear crystal is Fourier-imaged onto the object. This configuration is used in the experiments shown in Figure 4 of the manuscript and Figure 16. The two-photon field is written:

$$\Psi_t(x_1, x_2) = A'' \exp\left(\frac{-|x_1 + x_2|^2}{4\sigma_\theta^2 F^2}\right) \exp\left(\frac{-|x_1 - x_2|^2\sigma_r^2}{4\lambda_p^2 F^2}\right) \tag{14}$$

where A'' is an amplitude parameter and F is the effective focal length of the Fourier imaging system between the crystal output plane and the object plane. For a Fourier imaging system composed of $2r+1$ consecutive convergent lenses with focal lengths f_j ($j \in [1, 2r+1]$), placed at focal distance from each other, the effective focal length of the system is defined as $F = f_1 \frac{f_3 f_5 \cdots f_{2r+1}}{f_2 f_4 \cdots f_{2r}}$. In such a configuration, the momentum correlation width is the relevant width characterizing how strongly photon pairs are anti-correlated when they interact with the object and then imaged onto the camera.

2.4.2 Values of correlation widths in the camera plane

The correlation width in the camera plane is denoted σ. It corresponds to a position correlation width when using a near-field illumination, and to a momentum correlation width when using a far-field illumination. In practice, its value must be compared to the pixel width Δ to ensure that the imaging system is limited by the sensor resolution i.e. $\sigma < \Delta$ (necessary condition). As this value is difficult to measure in practice (precisely because we operate under this condition $\sigma < \Delta$), this quantity is estimated in each of our experiments using equations 11 and 12 and taking into account the magnifications in near-field configurations and effective focal length in far-field configurations.

Near-field illumination configuration Table 1 summarizes correlation width values at the output of the crystal and in the camera plane for all the experimental setups arranged in a near-field illumination configuration shown in the manuscript.

Far-field illumination configuration Table 2 summarizes correlation width values at the output of the crystal and in the camera plane for the different experimental setups arranged in a far-field illumination configuration shown in the manuscript.

Table 1

Experimental setup	Figure 1	Figure 3 / Figure 15	Figure 10
Pump wavelength λ_p	405 nm	405 nm	405 nm
Crystal thickness L	0.5 mm	0.5 mm	0.5 mm
Position correlation width at crystal output σ_{r_0}	$3.8\,\mu$m	$3.8\,\mu$m	$3.8\,\mu$m
Magnification between crystal and camera plane	3.3	2.1	3.3
Correlation width in camera plane σ	$13\,\mu$m	$8\,\mu$m	$13\,\mu$m

Table 2

Experimental setup	Figure 16	Figure 4
Pump wavelength λ_p	347nm	405nm
Crystal thickness L	0.5mm	0.5mm
Pump beam waist ω	0.5mm	0.8mm
Position correlation width at crystal output σ_{θ_0}	0.35mrad	0.25mrad
Effective focal length between crystal and camera plane	22.5mm	36mm
Correlation width in camera plane σ	$8\,\mu$m	9μm

2.5 Understanding JPD pixel super-resolution

In practice, a camera is an array of pixels with a finite pixel pitch Δ and gap δ, and photon pairs have a finite correlation width σ (Figure 2.a). To understand pixel super-resolution, we consider Eq. 9 that was obtained from Eq. 8 after applying the constraints (c1) and (c2).

Figure 2.b shows a graphical representation of Eq. 9. Using this representation, we analyze the specific roles played by the different elements in the integral i.e. the two-photon field $|\Psi_t(x_1, x_2)|^2$, the bi-variate function $|t(x_1)t(x_2)|^2$ and the integration areas $[x_k - \frac{\Delta-\delta}{2}, x_k + \frac{\Delta-\delta}{2}] \times [x_l - \frac{\Delta-\delta}{2}, x_l + \frac{\Delta-\delta}{2}]$:

- Integration areas $[x_k - \frac{\Delta-\delta}{2}, x_k + \frac{\Delta-\delta}{2}] \times [x_l - \frac{\Delta-\delta}{2}, x_l + \frac{\Delta-\delta}{2}]$ for different couple of integer (k, l) are represented by a grid of black squares in Figure 2.b of width Δ and spaced by δ. For clarify, we only represented 3 pixels i.e. 9 total black squares.

- The bi-variable function $|t(x_1)t(x_2)|^2$ is represented by a green area delimited by a green solid line.

- The two-photon wave-function $|\Psi_t(x_1, x_2)|^2$ is modelled by a double-gaussian, as detailed in the section 2.4.1. For clarity, we consider here the case of a near-field illumination configuration. The black dashed lines delimit an area so that $|x_2 - x_1| < \sigma$, which corresponds to the most intense part of the function $|\Psi_t(x_1, x_2)|^2$. Note also that, because of the constraint (c2), we have $\sigma < \Delta$.

The regions shown in blue and red in Figure 2.b are at the intersection of the grid, the bivariate function $|t(x_1)t(x_2)|^2$ and the wave-function $|\Psi_t(x_1, x_2)|^2$. They therefore are the most significant contributions to the integral in Eq. 9.

2.5.1 Blue regions, diagonal elements and sampling at x_k

Blue regions in Figure 2.b contribute to the diagonal elements Γ_{kk}. Importantly, we observe that each blue area is centred around integer positions (x_k, x_k). Thus, these JPD diagonal elements Γ_{kk} sample the object exactly at integer spatial positions x_k, similarly to what a camera measuring intensity in the conventional way would do. For small correlation width values $\sigma < \Delta$, it is clear on the Figure 2.b that the blue integration area is tightening around the position (x_k, x_k), which will results in $\Gamma_{kk} \sim |t(x_k)|^4$. More formally, one can also apply a change of variable and perform a first-order Taylor expansion in Eq. 9 to reach the same result. First, we apply the following change of variable:

$$x'_1 = -x_k + x_1 \tag{15}$$

$$x'_2 = -x_k + x_2 \tag{16}$$

For clarity, we also only consider $\delta \ll \Delta$. Equation 9 taken at $l = k$ simplifies into:

$$\Gamma_{kk} = \int_{-\Delta/2}^{\Delta/2} \int_{-\Delta/2}^{\Delta/2} |t(x_k + x'_1)t(x_k + x'_2)\Psi_t(x_k + x'_1, x_k + x'_2)|^2 dx'_1 dx'_2. \tag{17}$$

Then, we perform a first order Taylor expansion of the term $|t(x_k + x'_1)t(x_k + x'_2)|^2$:

$$|t(x_k + x'_1)t(x_k + x'_2)|^2 = |t(x_k)|^4 + [t(x_k)^2 t(x_k)^* \frac{dt}{dx}(x_k)^* + \text{cc}](x_2 + x_1) + o(\sqrt{x_1^2 + x_2^2}). \tag{18}$$

Inserting this Taylor expansion in Eq. 17 splits the integral in three terms:

$$\begin{aligned}\Gamma_{kk} &= |t(x_k)|^4 \int_{-\Delta/2}^{\Delta/2}\int_{-\Delta/2}^{\Delta/2} |\Psi_t(x_k+x_1, x_k+x_2)|^2 dx_1 dx_2 \\ &+ \quad [t(x_k)^2 t(x_k)^* \frac{dt}{dx}(x_k)^* + \text{cc}] \int_{-\Delta/2}^{\Delta/2}\int_{-\Delta/2}^{\Delta/2} |\Psi_t(x_k+x_1, x_k+x_2)|^2 (x_2+x_1) dx_1 dx_2 \\ &+ \quad \int_{-\Delta/2}^{\Delta/2}\int_{-\Delta/2}^{\Delta/2} |\Psi_t(x_k+x_1, x_k+x_2)|^2 o(\sqrt{x_1^2+x_2^2}) dx_1 dx_2. \end{aligned} \tag{19}$$

1. The first term in equation 19 can be written as $|t(x_k)|^4 S_0$, where:

$$S_0 = \int_{-\Delta/2}^{\Delta/2}\int_{-\Delta/2}^{\Delta/2} |\Psi_t(x_k+x_1, x_k+x_2)|^2 dx_1 dx_2. \tag{20}$$

S_0 can be calculated using the double Gaussian approximation provided in Eq. 13. However, the resulting expression includes some non analytical functions, such as the error function, that is not really useful for understanding it. To obtain an (approximative) analytical expression, we further approximate the Gauss function in Eq. 13:

- We use the fact that, in our experiment, pixel pitch value $\Delta \approx 10\mu m$ is much smaller than $\lambda_p/\sigma_\theta \approx 1mm$. In practice, this just reflects the fact that the area of illumination on the camera is much larger than the pixel pitch. Over the integration area $[0, \Delta] \times [0, \Delta]$, Eq. 13 simplifies as:

$$|\Psi_t(x_k+x_1, x_k+x_2)|^2 \approx e^{-\frac{(x_1-x_2)^2}{2\sigma^2}}. \tag{21}$$

- Because $\sigma < \Delta$, we further approximate the Gauss function in Eq. 21 by a square function:

$$e^{-\frac{(x_1-x_2)^2}{2\sigma^2}} \approx 1 \text{ if } |x_2-x_1| < \sigma, \text{ and } 0 \text{ otherwise.} \tag{22}$$

Under these approximations, we obtain a formula for S_0:

$$S_0 \approx \sigma/2[1 + 2\sqrt{2}(\Delta - \sigma/\sqrt{2})]. \tag{23}$$

2. The second term of equation 19 vanishes by symmetry when inserting Eq. 21.

3. The third term of equation 19 is a $o(\Delta^2)$, and therefore also a $o(\sigma^2)$.

Equation 17 can finally be written as:

$$\Gamma_{kk} = |t(x_k)|^4 S_0 + o(\sigma^2), \tag{24}$$

In addition, we also found also an (approximative) analytical form of $S_0 \approx \sigma/2[1 + 2\sqrt{2}(\Delta - \sigma/\sqrt{2})]$. Without assuming $\delta = 0$, this formula becomes: $S_0 \approx \sigma/2[1 + 2\sqrt{2}(\Delta - \delta - \sigma/\sqrt{2})]$.

2.5.2 Red regions, off-diagonal elements and sampling at $x_{k+1/2}$

Red regions in Figure 2.b contribute to the off-diagonal elements Γ_{kk+1}. Importantly, we observe that each red area is centered around half-integer positions $(x_{k+1/2}, x_{k+1/2})$. Thus, these JPD off-diagonal elements Γ_{kk+1} sample the object exactly at half-integer spatial positions x_{k+1}, similarly to what a camera shifted by half of the pixel pitch measuring intensity in the conventional way would do. For small correlation width values $\sigma < \Delta$, it is clear on the Figure 2.b that the red integration areas are tightening around the position $(x_{k+1/2}, x_{k+1/2})$, which will results in $\Gamma_{kk+1} \sim |t(x_{k+1/2})|^4$. More formally, one can also apply a change of variable and perform a first-order

Taylor expansion in Eq. 9 to reach the same result. First, we apply the following change of variable:

$$x_1' = \frac{\Delta}{2} + x_k - x_1 \quad (25)$$

$$x_2' = \frac{\Delta}{2} + x_k + x_2 \quad (26)$$

For clarity, we also only consider $\delta \ll \Delta$. Equation 9 taken at $l = k+1$ simplifies into:

$$\Gamma_{kk+1} = \int_0^\Delta \int_0^\Delta |t(x_{k+1/2} - x_1')t(x_{k+1/2} + x_2')\Psi_t(x_{k+1/2} - x_1', x_{k+1/2} + x_2')|^2 dx_1' dx_2'. \quad (27)$$

Then, we perform a first order Taylor expansion of the term $|t(x_{k+1/2} - x_1)t(x_{k+1/2} + x_2)|^2$:

$$|t(x_{k+1/2}-x_1)t(x_{k+1/2}+x_2)|^2 = |t(x_{k+1/2})|^4 + [t(x_{k+1/2})^2 t(x_{k+1/2})^* \frac{dt}{dx}(x_{k+1/2})^* + \text{cc}](x_2 - x_1) + o(\sqrt{x_1^2 + x_2^2}), \quad (28)$$

Inserting this Taylor expansion in Eq. 27 splits the integral in three terms:

$$\begin{aligned} \Gamma_{kk+1} &= |t(x_{k+1/2})|^4 \int_0^\Delta \int_0^\Delta |\Psi_t(x_{k+1/2} - x_1, x_{k+1/2} + x_2)|^2 dx_1 dx_2 \\ &+ [t(x_{k+1/2})^2 t(x_{k+1/2})^* \frac{dt}{dx}(x_{k+1/2})^* + \text{cc}] \int_0^\Delta \int_0^\Delta |\Psi_t(x_{k+1/2} - x_1, x_{k+1/2} + x_2)|^2 (x_2 - x_1) dx_1 dx_2 \\ &+ \int_0^\Delta \int_0^\Delta |\Psi_t(x_{k+1/2} - x_1, x_{k+1/2} + x_2)|^2 o(\sqrt{x_1^2 + x_2^2}) dx_1 dx_2. \end{aligned} \quad (29)$$

1. The first term in equation 29 can be written as $t(x_{k+1/2})^4 S_1$, where:

   $$S_1 = \int_0^\Delta \int_0^\Delta |\Psi_t(x_{k+1/2} - x_1, x_{k+1/2} + x_2)|^2 dx_1 dx_2. \quad (30)$$

   S_1 can be calculated using the double Gaussian approximation provided in Eq. 13. However, the resulting expression includes some non analytical functions, such as the error function, that is not really useful for understanding it. To obtain an (approximative) analytical expression, we further approximate the Gauss function in Eq. 13:

- We use the fact that, in our experiment, pixel pitch value $\Delta \approx 10\mu m$ is much smaller than $\lambda_p/\sigma_\theta \approx 1mm$. In practice, this just reflects the fact that the area of illumination on the camera is much larger than the pixel pitch. Over the integration area $[0,\Delta] \times [0,\Delta]$, Eq. 13 simplifies as:

$$|\Psi_t(x_{k+1/2} - x_1, x_{k+1/2} + x_2)|^2 \approx e^{-\frac{(x_1+x_2)^2}{2\sigma^2}}. \tag{31}$$

- Because $\sigma < \Delta$, we further approximate the Gauss function in Eq. 31 by a square function:

$$e^{-\frac{(x_1+x_2)^2}{2\sigma^2}} \approx 1 \text{ if } |x_2 + x_1| < \sigma, \text{ and } 0 \text{otherwise.} \tag{32}$$

Under these approximations, we obtain a formula for S_1:

$$S_1 \approx \frac{\sigma^2}{2}. \tag{33}$$

2. The second term of equation 29 vanishes by symmetry when inserting Eq. 31.

3. The third term of equation 29 is a $o(\Delta^2)$, and therefore also a $o(\sigma^2)$.

Equation 27 can finally be written as:

$$\Gamma_{kk+1} = |t(x_{k+1/2})|^4 S_1 + o(\sigma^2), \tag{34}$$

In addition, we also found also an (approximative) analytical form of $S_1 \approx \sigma^2/2$. Without assuming $\delta = 0$, this formula becomes: $S_1 \approx (\sigma - \delta\sqrt{2})^2/2$.

2.5.3 Case of far-field illumination configuration

The above reasoning applies directly to the far-field illumination configuration. In this case, Ψ_t takes the form described by Eq. 14. Anti-diagonal elements $\Gamma_{k\,-k}$ sample the object at integer spatial positions x_k, and the off-anti-diagonal elements $\Gamma_{k\,-k+1}$ samples the object exactly at half-integer spatial positions $x_{k+1/2}$. The corresponding values are then interlaced using a minus-coordinate projection of the JPD (Eq. 6) after the same normalisation process. Note that, in such a configuration an object is generally positioned in only one half of the beam ($t(x) = 0$ if $x < 0$), meaning that the resulting image contains two copies of the pixel super-resolved image, but no information is lost.

2.5.4 Normalization and sum-coordinate projection

Measuring the JPD provides two different images: (i) a diagonal image formed by the values Γ_{kk}, which correspond to a sampling of the object on integer positions x_k, and (ii) a off-diagonal image formed by the values Γ_{kk+1} which corresponds to a sampling of the object on half-integer positions $x_{k+1/2}$. The values of these two images should be interlaced to obtain a higher resolution image. This is precisely the task performed by the sum-coordinate projection of the JPD i.e. Eq. 4. But before this projection, each image must be normalised to compensate the difference in integration area $S_1 \neq S_2$. In our work, such a normalisation is achieved by dividing the values in each image by the sum of all its values i.e. $\Gamma_{kk} \rightarrow \Gamma_{kk}/\sum_k \Gamma_{kk}$ and $\Gamma_{kk+1} \rightarrow \Gamma_{kk+1}/\sum_k \Gamma_{kk+1}$. Indeed, in

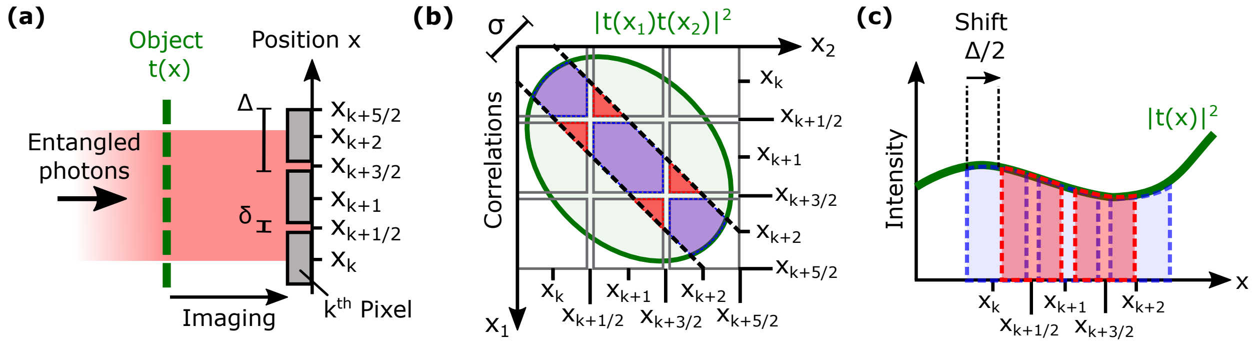

Supplementary Figure 2: ***Principle of JPD pixel super-resolution.*** *n band of photon-pairs with correlation width σ. Green lines delimit the bi-variate function $|t(x_1)t(x_2)|^2$. The correlation band and the bi-variate function overlap with diagonal pixels at the blue areas and with off-diagonal pixels at the red areas.* ***(c)*** *Schematic of classical shift-and-add approach. Blue areas under the curve $|t(x)|^2$ represent direct intensity values measured by three pixels centred at integer positions x_k, x_{k+1} and x_{k+2}. Red areas are intensity values measured after shifting the sensor by half a pixel in the transverse direction and centred at half-integer positions $x_{k+1/2}$ and $x_{k+3/2}$. A high-resolution image is obtained by combining the two images and enables pixel super-resolution.*

a good approximation the sum of all the image values is proportional to the respective integration area S_0 or S_1.

Note that, in some case, such as the experiment with the SPAD camera shown in Figure 16.a, such a normalisation procedure is not precise enough because the difference between S_1 and S_2 is too large. To solve this issue, more advanced algorithms such as L_1 or L_2 norms minimisation approaches [8] and kernel regression [9] can be used to improve the process and eliminate the remaining artefacts in the high-resolution image.

2.6 Analogy with shift-and-add

Our approach is the analogue of the shift-and-add technique used in classical imaging [10]. Figure 2.c illustrates this classical process. First, a conventional intensity image is acquired by positioning the camera so that pixels are centred on integer spatial positions x_k. Then, the camera is shifted by half of a pixel along the transverse axis to center pixels on half-integer spatial positions $x_{k+1/2}$ and a second intensity image is acquired. Finally, a high-resolution image is obtained by interlacing values of these two images. The main difference between JPD pixel super-resolution and shift-and-add is that the integration areas are different for each low-resolution image in the case of JPD ($S_0 \neq S_1$), whereas they remain identical in the case of shift-and-add. This small difference will influence the shape of the corresponding Modulation Transfer Function (MTF) measured when using the slanted edge approach (see section 4).

2.7 Qualitative simulations to illustrate the JPD pixel super-resolution technique

Near-field illumination configuration. Figure 3 shows a 1D simulation illustrating the principle of our technique when using a near-field illumination configuration. In such a simple imaging configuration, described in Figure 3.a, a thin type-I BBO crystal is illuminated by a collimated blue pump laser to produce spatially-entangled photon pairs. Crystal surface, object (grid-shape) and camera are positioned in the same optical plane. In this simulation, photon-pairs are considered to be strongly correlated in space. Figure 3.b shows the JPD of photon-pairs measured without the object. The JPD is 2D-matrix because the imaging system is 1D. The intense diagonal visible in the JPD is a clear signature of the near-perfect position correlations between photon pairs. After inserting the object, the diagonal of the JPD becomes modulated by the object shape (Figure 3.c). Figure 3.d shows the JPD after pixellation (with $\sigma < \Delta$). Figure 3.e shows the JPD sum-coordinate projection, obtained by summing correlation values along the anti-diagonals. This projection enables to retrieve a pixel super-resolved image of the object. Figure 3.f shows the JPD minus-coordinate projection, obtained by summing correlation values along the diagonals. This projection shows an intensity correlation peak that is a clear signature of photon correlations.

Far-field illumination configuration. Figure 4 shows a 1D simulation illustrating the principle of our technique when using a far-field illumination configuration. In this simple configuration, described in Figure 4.a, a thin type-I BBO crystal is illuminated by a collimated blue pump laser

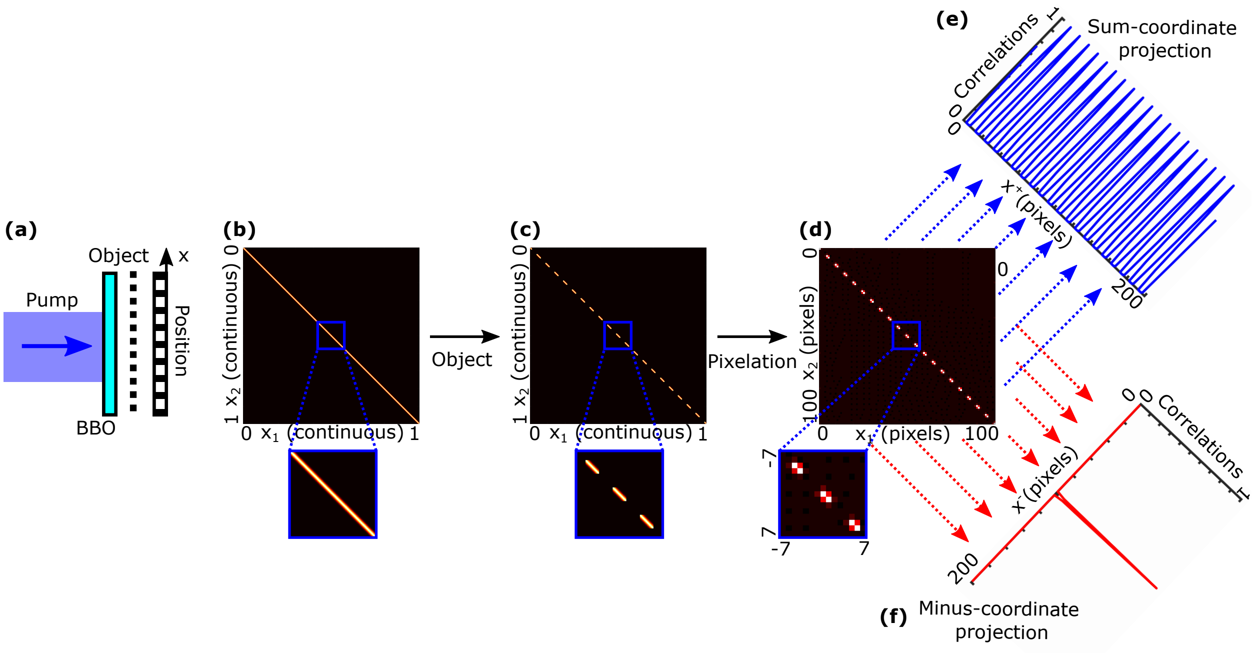

Supplementary Figure 3: ***Simple illustration of JPD pixel super-resolution using a near-filed illumination configuration.*** ***(a)*** *1D scheme for imaging with spatially-entangled photon pairs using a near-field illumination configuration. Crystal surface, object and camera are positioned in the same optical plane.* ***(b)*** *JPD without object.* ***(c)*** *JPD with object.* ***(d)*** *JPD with object after pixellation. In this image, the colorbar is intentionally saturated to make the off-diagonal coefficients well visible. The real ratio between the diagonal and off-diagonal coefficients is about 10.* ***(e)*** *Sum-coordinate projection of the JPD.* ***(f)*** *Minus-coordinate projection of the JPD.*

to produce spatially-entangled photon pairs. The crystal surface is Fourier-imaged onto the object, that is imaged onto the camera. In this simulation, photon-pairs are considered to be strongly anti-correlated. The object has a grid shape and is positioned in only half of the optical plane (the other half is empty). Figure 4.b shows the JPD of photon-pairs measured without the object. The JPD is a 2D-matrix because the imaging system is 1D. The intense anti-diagonal visible in the JPD is a clear signature of the strong anti-correlations between photon-pairs. After inserting the object, we observe that each half of the JPD anti-diagonal become modulated by the object shape (Figure 4.d). Figure 3.d shows the JPD after pixellation (with $\sigma < \Delta$). Figure 3.e shows the JPD sum-coordinate projection, obtained by summing correlation values along the anti-diagonals. This projection shows a intensity correlation peak that is a clear signature of anti-correlation between photon pairs. Figure 3.f shows the JPD sum-coordinate projection, obtained by summing correlation values along the diagonals. This projection enables to retrieve a pixel super-resolved image proportional to $|t(x/2)||t(-x/2)|^2$. Because the object is positioned in only one half of the beam, the resulting image contains two copies of the pixel super-resolved image.

2.8 Quantitative simulations of JPD pixel super-resolution.

Figure 5 and 6 show simulation results demonstrating the pixel super-resolution effect. The main steps of the algorithm to obtain these results are described in the following:

1. *Two-photon input field.* A two-photon wave-function was produced using the Gaussian

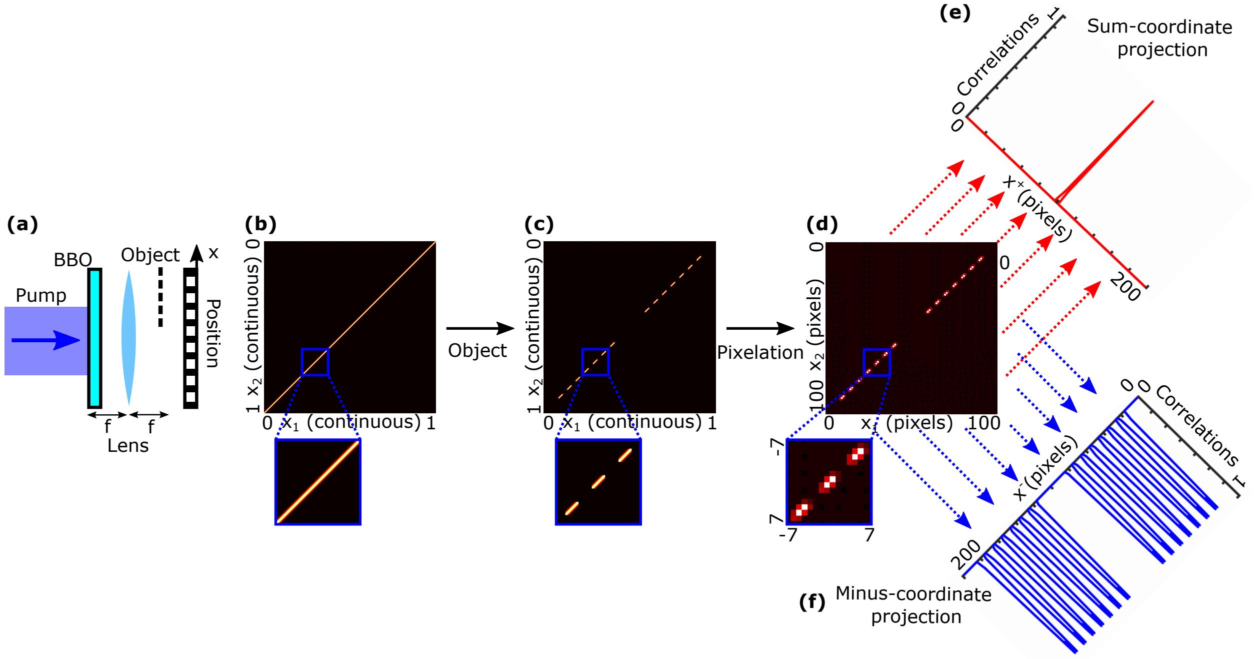

Supplementary Figure 4: ***Simple illustration of JPD pixel super-resolution using a far-filed illumination configuration.*** ***(a)*** *1D scheme for imaging with spatially-entangled photon pairs using a far-field illumination configuration. Crystal surface is Fourier-imaged onto the object positioned in one half of the optical plane. Object is imaged on the camera.* ***(b)*** *JPD without object.* ***(c)*** *JPD with object.* ***(d)*** *JPD with object after pixellation. In this image, the colorbar is intentionally saturated to make the off-diagonal coefficients well visible. The real ratio between the diagonal and off-diagonal coefficients is about 10.* ***(e)*** *Sum-coordinate projection of the JPD.* ***(f)*** *Minus-coordinate projection of the JPD.*

model in Eq. 13 (i.e. near-field illumination configuration). In Matlab, such a function takes the form of a 1000×1000 pixels complex matrix noted Ψ_{in}. Correlation widths are defined relative to the pixel size: $\sigma_r = \sigma = 3.5$ and $\sigma_k = 1.4e{-}6\,\lambda_p$.

2. *Object.* In this simulation, we chose an object with a spectrum that has a simple shape: a sine-shaped object i.e. $t(x) = \sin(2\pi f x)$, where x is a spatial vector and f the frequency that we vary in the simulation. In Matlab, t takes the form of a vector with 1000 pixels.

3. *Propagation.* The two-photon field after propagation through the object is obtained using Eq. 7 under the approximation $h(x', x) = \delta(x' - x)$ (i.e. constraint (c1)). In Matlab, the double-integral in Eq. 7 is calculated by performing the following matrix multiplication:

$$\Psi_{out} = \text{diag}(t)\Psi_{in}\text{diag}(t)^T \tag{35}$$

where Ψ_{out} is the matrix associated with the two-photon output field and $\text{diag}(t)$ is a diagonal matrix containing the vector-object t on its diagonal. The corresponding joint probability distribution is noted $\Gamma_{out} = |\Psi_{out}|^2$.

4. *Pixelation.* As described above, the object and the joint probability distribution are sampled over 1000 pixels, which allows to resolve the images well. To simulate the effect of pixelation, pixel values in the output images are grouped into macro pixels, which is similar to what a camera does in practice. In Matlab, this is done simply by multiplying the output joint probability distribution Γ_{out} on each side by a pixelation matrix P_L:

$$\Gamma_{out}^{pix} = P_\Delta \Gamma_{out} P_\Delta^T, \tag{36}$$

where Γ_{out}^{pix} is the joint probability distribution measured after pixelation. In equation. 36, P_Δ is a mathematical tool that enables to quickly group the values of the 1000 pixels into $1000/\Delta$ macro pixels i.e. each macro pixel is composed of Δ pixels. It is defined as a matrix of size $(1000/\Delta) \times 1000$ composed of only 0 and 1: for all $i \in [1, \Delta]$ (row) and $j \in [1, 1000]$ (column), $P_\Delta(i,j) = 1$ if $j \in [i, i+\Delta]$, and 0 otherwise. Similarly, the classical intensity image I_{out}^{pix} produced at the output after pixelation is obtained by multiplying the vector-object by the pixelation matrix:

$$I_{out}^{pix} = P_\Delta |t|^2. \tag{37}$$

In our simulation, we chose a pixelation parameter of $\Delta = 10$, which means that each macro pixel has a size of 10 pixels. This choice is not made at random: it ensures that the correlation width σ is smaller than that of a pixel Δ (constraint (c2)), with a ratio of $\sigma/\Delta = 0.35$ that is similar to the one we have in our experiments. For example, in the experiment shown in Figure 1.a of the manuscript, the ratio $\sigma/\Delta = 13/32 \approx 0.4$ (see also tables 1 and 2). In our simulation, we assumed a perfect fill factor i.e. $\delta = 0$ for clarity.

In this simulation, five different types of images are produced at the output for four different values of $f \in [0.065, 0.22, 0.27, 0.4]$ (frequency of the sine-shaped object, normalized to the sampling frequency $1/\Delta$). All these images are shown in Figure 5:

- Classical intensity images without pixelation (i.e. just $|t|^2$) are shown in Figure 5.1a.1b.1c.1d for the four different frequencies $f \in [0.065, 0.22, 0.27, 0.4]$. These high-resolution images (1000 pixels) do not correspond to any experimental output. They are shown here as ground-

truth images to better appreciate the deformation created by the pixelation effect.

- Classical intensity images with pixelation $\Delta = 10$ are shown in Figure 5.2a.2b.2c.2d for the four different frequencies $f \in [0.065, 0.22, 0.27, 0.4]$.
- JPD diagonal images with pixelation $\Delta = 10$ are shown in Figure 5.3a.3b.3c.3d for the four different frequencies $f \in [0.065, 0.22, 0.27, 0.4]$.
- JPD sum-coordinate projection with pixelation $\Delta = 10$ are shown in Figure 5.4a.4b.4c.4d for the four different frequencies $f \in [0.065, 0.22, 0.27, 0.4]$.
- Classical intensity images with pixelation $\Delta' = \Delta/2 = 5$, which simulate the intensity images obtained using with a half-pixel-pitch camera, are shown in Figure 5.5a.5b.5c.5d for the four different frequencies $f \in [0.065, 0.22, 0.27, 0.4]$.

To compare these images quantitatively, we use spectral representations obtained by Fourier transform shown in Figure 6. First of all, it is important to note that the Fourier spectrum of a sine-shaped of frequency f squared contains two components: (i) a peak at 0 and (ii) a peak at $2f$. In our analysis, we vary the frequency of the $2f$-peak by varying f. For $f = 0.065$ (Figure 6.a) and $f = 0.022$ (Figure 6.a), values of the $2f$-peak (i.e. $2f = 0.13$ and $2f = 0.44$, respectively) are smaller than the Nyquist frequency of the sensor (0.5), and therefore the corresponding spectra are not altered by pixelation: all spectral curves are superimposed. The pixelation effect becomes clearly visible in Figure 6.c where $2f = 0.54$ that is greater than the sensor Nyquist frequency. In this figure, we observe two very distinct behaviours of the curves: the $2f$-peaks in the intensity (red curve) and JPD diagonal (black dashed-curve) curves are reflected on the left with respect

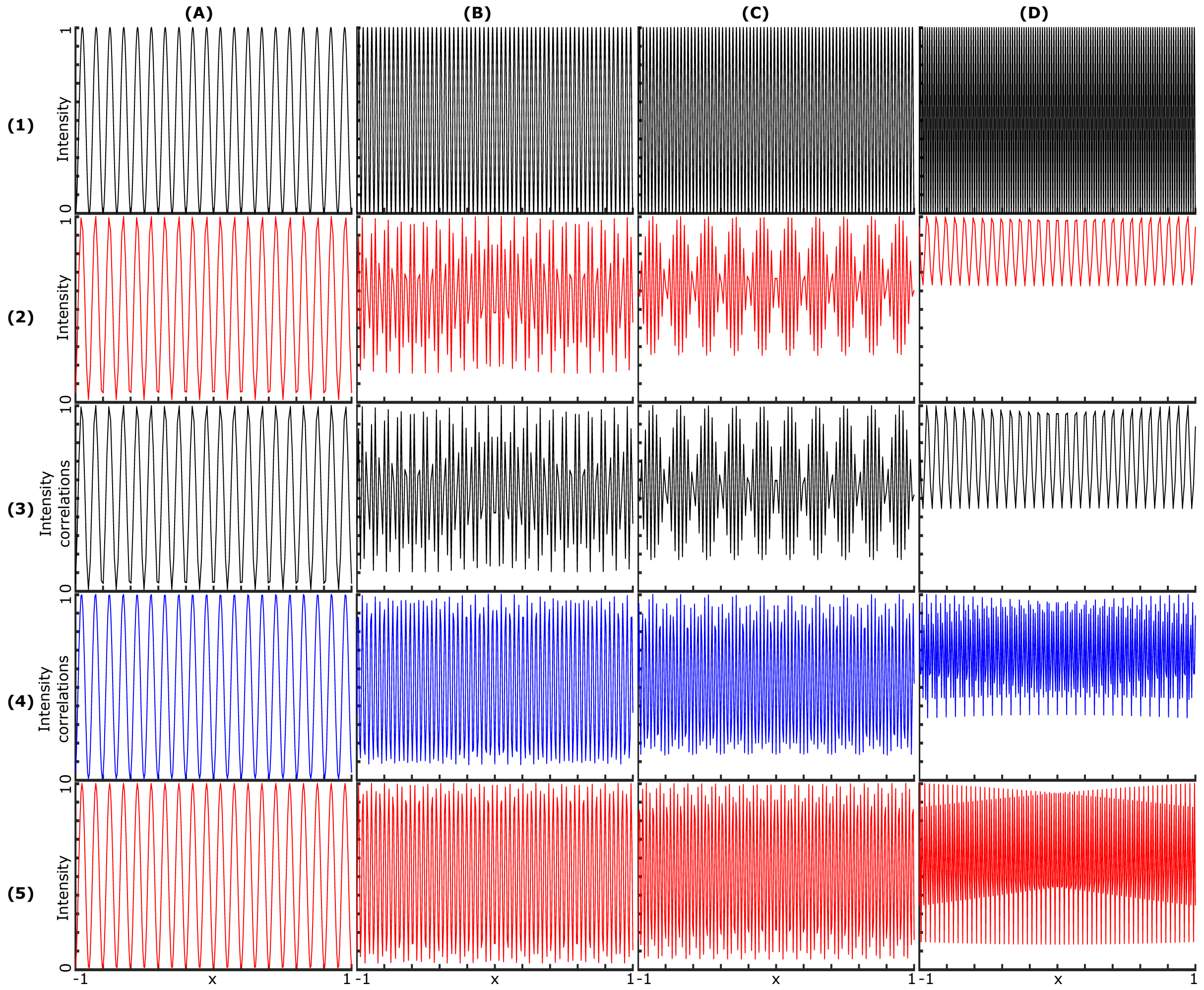

Supplementary Figure 5: ***Quantitative simulation: output images.*** *The output images are represented in the figure as a matrix of images. Each line is identified by a number* ***(1-5)*** *and corresponds to the type of image considered:* ***(1)*** *Classical intensity image without pixelation,* ***(2)*** *classical intensity image with pixelation* $\Delta = 10$*,* ***(3)*** *JPD diagonal image,* ***(4)*** *JPD sum-coordinate projection and* ***(5)*** *classical intensity image with half-pixel-pitch sensor i.e. pixelation* $\Delta' = 5$*. Each column is identified by a letter* ***(a-d)*** *and corresponds to a certain frequency of the sine-shaped object:* ***(a)*** $f = 0.065$*,* ***(b)*** $f = 0.22$*,* ***(c)*** $f = 0.27$ *and* ***(d)*** $f = 0.4$*.*

to the Nyquist limit and positioned at $0.5 - (0.54 - 0.5) = 0.46$, while the $2f$-peaks in the JPD sum-coordinate projection (blue dashed-curve) and intensity curve obtained with a sensor of half-pixel-pitch (red dashed-curve) are correctly positioned at 0.54. This reflection of the $2f$-peaks with respect to the Nyquist limit is the aliasing effect. It is present in the intensity and JPD diagonal images, but absent in the JPD sum-coordinate projection. This shows that measuring the JPD sum-coordinate projection achieves anti-aliasing and enables to retrieve an image that is similar to the one that would be obtained with a camera with a half-pixel-pitch i.e. containing four times more pixels. The effect is even more pronounced when programming a higher frequency $2f = 0.8$, as shown in Figure 6.d. The supplementary video [11] shows an animation of the spectra in which the frequency f is varied continuously from 0 to 1.

2.9 Generalization to a two-dimensional discrete space

This one-dimensional analysis is easily generalized to the real case of two-dimensional objects and cameras. In the 2D case, the JPD is a four-dimensional matrix Γ_{ijkl}. It contains 1 diagonal image Γ_{ijij} and 8 off-diagonal images $\Gamma_{ij\,i\pm1\,j}$, $\Gamma_{ij\,i\,\pm1}$ and $\Gamma_{ij\,i\pm1\,j\pm1}$, and the high-resolution image is obtained by performing the general sum- or minus-coordinate projection described by Equations 1 and 3 of the manuscript.

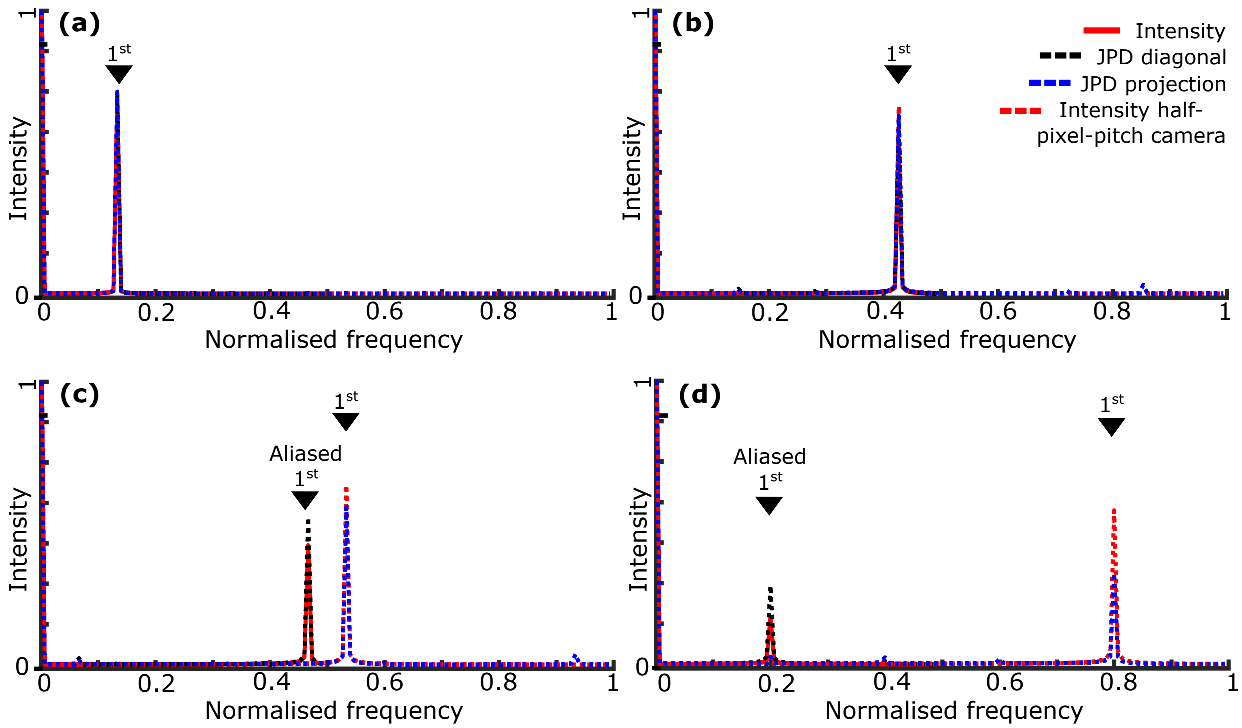

Supplementary Figure 6: ***Quantitative simulation: spectral analysis.*** *Each spectrum* ***(a-d)*** *shows 4 spectral curves which are obtained by Fourier transform of the different output images. Red curves correspond to classical intensity images with pixelation ($\Delta = 10$), black dashed-curves correspond to JPD diagonal images, blue dashed-curves correspond to JPD sum-coordinate projections and red dashed-curves correspond to classical intensity images obtained with a half-pixel-pitch sensor (i.e. pixelation $\Delta' = 5$). Spectral curves in* ***(a)***, ***(b)***, ***(c)*** *and* ***(d)*** *are obtained using a sine-shaped object of frequency $f = 0.065$, $f = 0.22$, $f = 0.27$ and $f = 0.4$, respectively.*

3 Additional measurements using a 48μm-pixel-pitch camera

Figure 7 shows results that complement those in Figure 1 of the manuscript. To obtain them, the same experiment as shown in Figure 1.a of the manuscript was used, but replacing the camera with an even lower resolution camera with a pixel pitch of 48μm. As expected, the intensity image shown in Figure 7.a is of very poor quality and does not reflect the characteristics of the object being imaged. In particular, the frequency at 0.4 is completely absent from the spectrum (solid red line in Figure 7.e) as it is higher than the sensor Nyquist frequency. It is replaced by a peak at 0.26 that is an aliased frequency. The acquisition of a JPD diagonal image shown in Figure 7.b worsens this problem: the image obtained has a higher contrast which increases the aliasing effect (more intense first order peak in the black dashed curve in Figure 7.e). The use of the JPD super-resolution technique, on the other hand, results in an image shown in Figure 7.c that is less altered and more similar to the object. In particular, our approach removes the aliasing effect and recovers the fundamental peak at 0.4, as seen in the corresponding spectrum in Figure 7.e (blue dashed line). To compare these results, an intensity image obtained with a higher resolution camera (16μm pixel-pitch) is shown in Figure 7.d and its spectrum in Figure 7.e (red dashed line).

4 Slanted-edge approach

The modulation transfer function (MTF) shown in Figure 1.g of the manuscript is obtained using the slanted edge technique [12]. Slanted edge is a standard technique in imaging. It allows an accurate

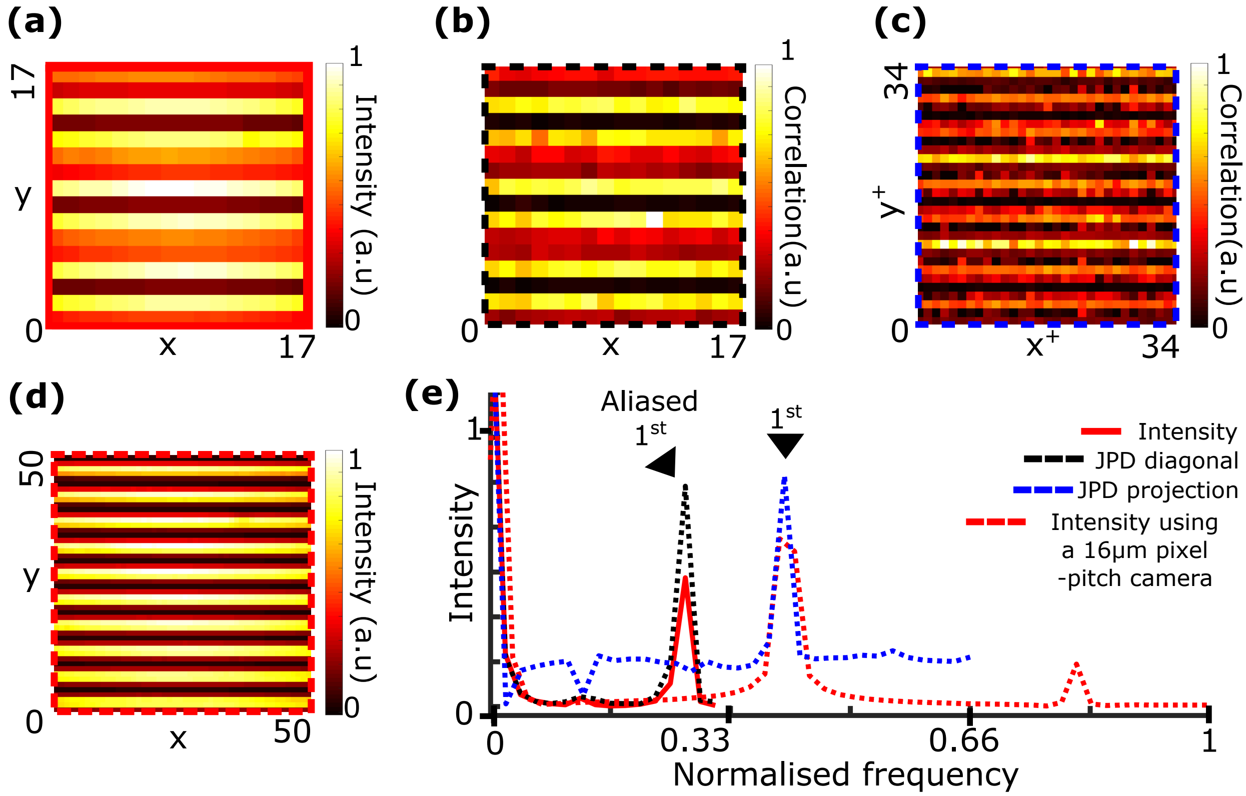

Supplementary Figure 7: ***Complementary demonstration experiment of JPD super-resolution.*** *The experimental setup used here is the same than this shown in Figure 1.a of the manuscript after replacing the camera by a lower resolution camera with* $48\mu m$ *pixel-pitch.* ***(a)*** *Intensity image,* ***(b)*** *JPD diagonal image and* ***(c)*** *sum-coordinate projection of the JPD.* ***(e)*** *Intensity image obtained using a camera with a smaller pixel pitch i.e.* $16\mu m$*. All images show the same spatial region of the object containing* 10 *grating periods. Coordinate are in pixels.* ***(f)*** *Spectra of the intensity image (solid red), diagonal image (dashed black), sum-coordinate image (dashed blue) and intensity image acquired with a* $16\mu m$*-pixel-pitch camera (dashed red) obtained by performing a discrete Fourier transform to the corresponding image and averaging aver the* x*-axis. All frequency values are normalized to the same reference frequency* $k_0 = 1/32\,\mu m^{-1}$.

measurement of the MTF of the imaging system without the problem of sampling and therefore of spectrum folding. The width of the curve obtained is the essential criterion for quantifying the bandwidth of the optical system and therefore its spatial resolution. In our experiment, the standard value MTF50, defined as the frequency at which the curve is 50% of its maximum, is used to compare the different curves measured. The larger this value, the higher the bandwidth of the imaging system and the higher its spatial resolution.

4.1 Description of the technique

To implement this technique, we insert an edge in the object plane of our imaging system and tilt it by a few degrees (here approximately 100mrad). We then measure a conventional intensity image (Fig. 8.a), a diagonal image (Fig. 8.b) and a projection (Fig. 8.c). To compare our results, we also measured an intensity image with a higher resolution camera i.e. 16μm pixel-pitch (Fig. 8.d). The MTF is then computed from each of these images using the algorithm detailed in [12].

4.2 Results interpretation

In theory, the spectral width of the MTF measured is inversely proportional to the optical resolution of the imaging system i.e. $\sim 1/r$ where r is the spatial resolution, and also inversely proportional to the pixel size of the camera i.e. $\sim 1/(\Delta - \delta)$. In our experiment shown in Figure 1.a, the imaging system is not diffraction limited and therefore the measured MTF width (Figure 1.e of

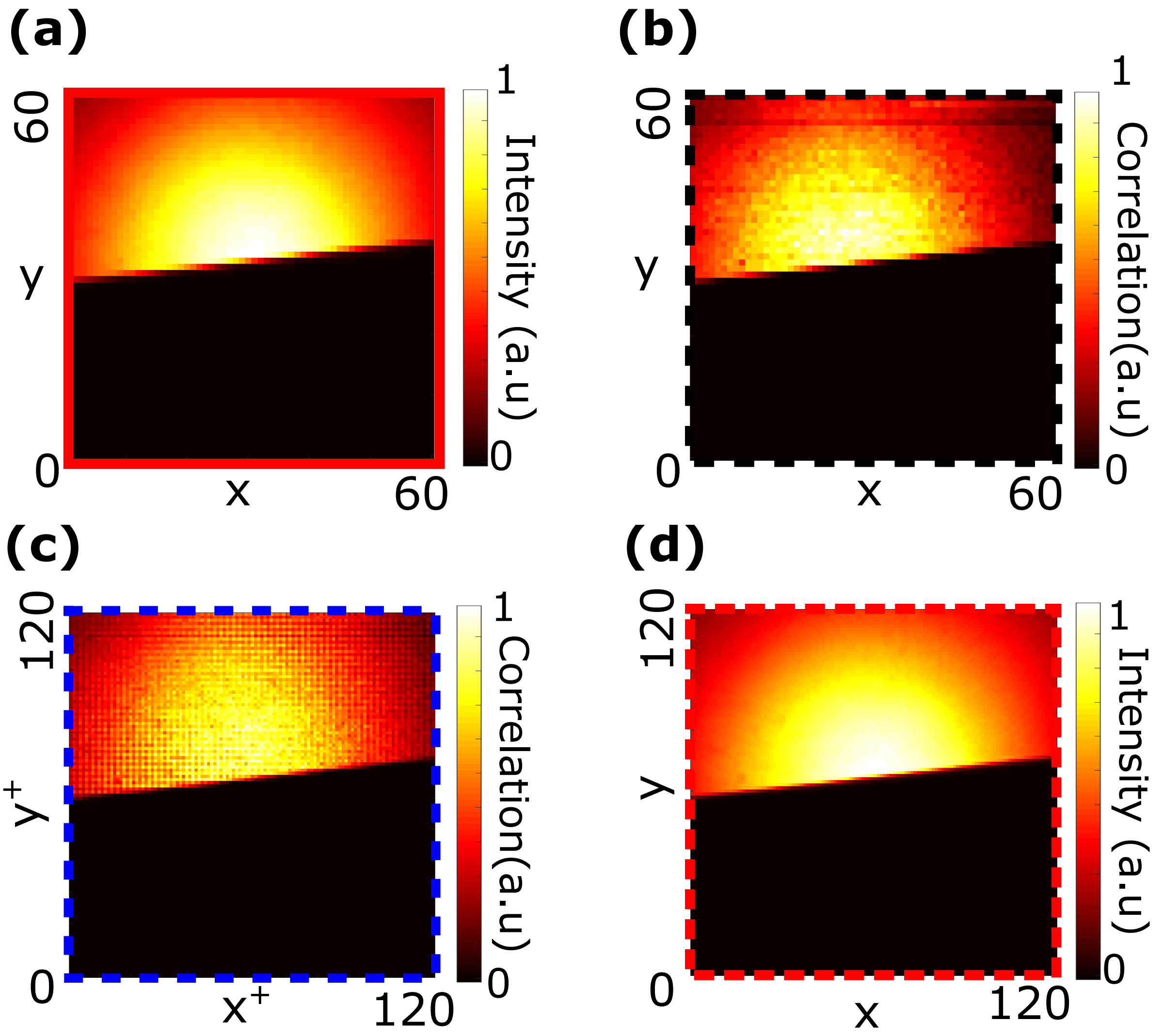

Supplementary Figure 8: ***Images acquired to implement the slanted edge technique.*** *An edge is inserted in the object plane of the experiment described in Figure 1.a of the manuscript and tilted by approximately* 100*mrad.* ***(a)*** *Intensity image,* ***(b)*** *JPD diagonal image and* ***(c)*** *sum-coordinate projection of the JPD.* ***(e)*** *Intensity image obtained using a camera with a smaller pixel pitch i.e.* 16μm*. Coordinate are in pixels.*

the manuscript) depends almost exclusively on the pixel width i.e. $1/(\Delta - \delta) \ll 1/r$. This can be seen by comparing the MTF measured with cameras with 32μm and 16μm pixel width: the bandwidth obtained with the camera with 16μm pixel width (dashed red line in Figure 1.e of the manuscript) is almost exactly twice as wide as the 32μm pixel width (solid red line in Figure 1.e of the manuscript).

It should be noted that the comparison of MTFs in Figure 1.e of the manuscript shows another improvement in spatial resolution obtained by our technique in addition to the one based on the increase in Nyquist frequency. Indeed, this analysis shows that the MTF of the JPD super-resolved image is wider independently of the Nyquist frequency. It is as if the camera not only had four times as many pixels, but also as if it had smaller pixels.

This reduction in effective pixel size follows directly from the analysis given in section 2, where we showed that the high resolution image is obtained by interlacing the values from two different types of image: (i) a diagonal image and a (ii) off-diagonal image. However, unlike the classical shift-and-add technique for example, these two images are not obtained from the same integration areas i.e. $S_0 \neq S_1$. In particular, the S_1 surface does not depend on the pixel size but only on the correlation width of the photons and the gap between the pixels. It is necessarily smaller than S_0 because we are working under the constraint (c2) which imposes $\sigma < \Delta$. Thus, the effective pixel size used to measure the off-diagonal image is smaller than the true width of a pixel. The high-resolution image is therefore retrieved using effective pixels that are on average smaller than the true width of a camera pixel, which leads to the widening of the MTF seen in Figure 1.e

of the manuscript.

5 Details on entanglement-enabled quantum holography

This section provides more details about the entangled-enabled quantum holography protocol used in Figure 4.a of the manuscript. This protocol has been introduced in [13].

5.1 Theory

Assuming that photon-pairs are near-perfectly correlated in space [6], the vectorial two-photon wave-function $\mathbf{\Psi}$ in the object/SLM plane simplifies as:

$$\mathbf{\Psi}(\mathbf{r_1}, \mathbf{r_2}) = \delta(\mathbf{r_1} + \mathbf{r_2}) \left[t_6^H(\mathbf{r_1}) t_6^H(\mathbf{r_2}) \mathbf{e_H} + e^{i(\alpha(\mathbf{r_1}) + \alpha(\mathbf{r_2}))} t_6^V(\mathbf{r_1}) t_6^V(\mathbf{r_2}) \mathbf{e_V} \right] \tag{38}$$

where t_6^H (resp. t_6^V) is the horizontal (resp. vertical) polarization component of the polarisation sensitive object t_6, $\mathbf{e_H}$ and $\mathbf{e_V}$ are the unit horizontal and vertical polarisation vectors, δ is the Dirac delta function and $\alpha(\mathbf{r})$ is the phase programmed at position $\mathbf{r}$ of the SLM. In the results shown in Figure 4 of the manuscript, t_6 is a birefringent object i.e. $t_6^H = |t_6| e^{\theta_H}$ and $t_6^V = |t_6| e^{\theta_V}$, where θ_H and θ_V are the horizontal and vertical polarization components of the object phase, respectively. As a result, after passing through the polariser at $45°$, the JPD measured by the camera takes the form of :

$$\Gamma(\mathbf{r_1}, \mathbf{r_2}) = |t_6(\mathbf{r_1}) t_6(\mathbf{r_2}) \delta(\mathbf{r_1} + \mathbf{r_2}) \left[1 + \cos\left(\Delta\theta(\mathbf{r_1}) + \Delta\theta(\mathbf{r_2}) - \alpha(\mathbf{r_1}) - \alpha(\mathbf{r_2})\right)\right]|^2 \tag{39}$$

where $\Delta\theta(\mathbf{r}) = \theta_H(\mathbf{r}) - \theta_V(\mathbf{r})$ is the object spatial phase that we are trying to retrieve.

5.2 Phase image retrieved using JPD anti-diagonals $\Gamma(\mathbf{r}, -\mathbf{r})$ (no pixel super-resolution)

To achieve quantum holography using the conventional protocol described in [13], only the anti-diagonal component of the JPD $\Gamma(\mathbf{r}, -\mathbf{r})$ needs to be considered. Taking into account that in our experiment (Figure 4.a of the manuscript) the object t_6 and the SLM are positioned on each halves of the optical plane (i.e. $t_6(x < 0, y) = 1$ and $\alpha(x > 0, y) = 0$), the JPD diagonal measured by the camera then simplifies as:

$$\Gamma(\mathbf{r}, -\mathbf{r}) = |t_6(\mathbf{r})|^2 \left[1 + \cos(\Delta\theta(-\mathbf{r}) - \alpha(\mathbf{r}))\right]^2 \text{ , for all } \mathbf{r} = (x > 0, y) \tag{40}$$

In the holographic reconstruction process, four JPD anti-diagonals (noted $\Gamma_\alpha(\mathbf{r}, -\mathbf{r})$) are measured for four different uniform phase shifts $\alpha(\mathbf{r}) \in \{0, \pi/2, \pi, 3\pi/2\}$ programmed on the SLM. Figures 9.c-f show the four anti-diagonal images measured at each phase value. The spatial phase $\Delta\theta(\mathbf{r})$ shown in Figure 9.k (and also in Figure 4.b of the manuscript) is then reconstructed from these four JPD anti-diagonals using the formula: $\arg[\Gamma_0(\mathbf{r}, -\mathbf{r}) - \Gamma_\pi(\mathbf{r}, -\mathbf{r}) + i(\Gamma_{\pi/2}(\mathbf{r}, -\mathbf{r}) - \Gamma_{3\pi/2}(\mathbf{r}, -\mathbf{r}))] = \Delta\theta(\mathbf{r})$. In practice, note also that a static and non-uniform phase distortion term (noted $\Phi_0(\mathbf{r})$) is also present in Equation 40, originating from the photon generation process [14]. This term is not shown here for clarify because it is directly compensated in the experiment by superimposing a correction phase pattern $-\Phi_0(\mathbf{r})$ to all the uniform phase α programmed on the SLM (see [13] for more details about the phase correction).

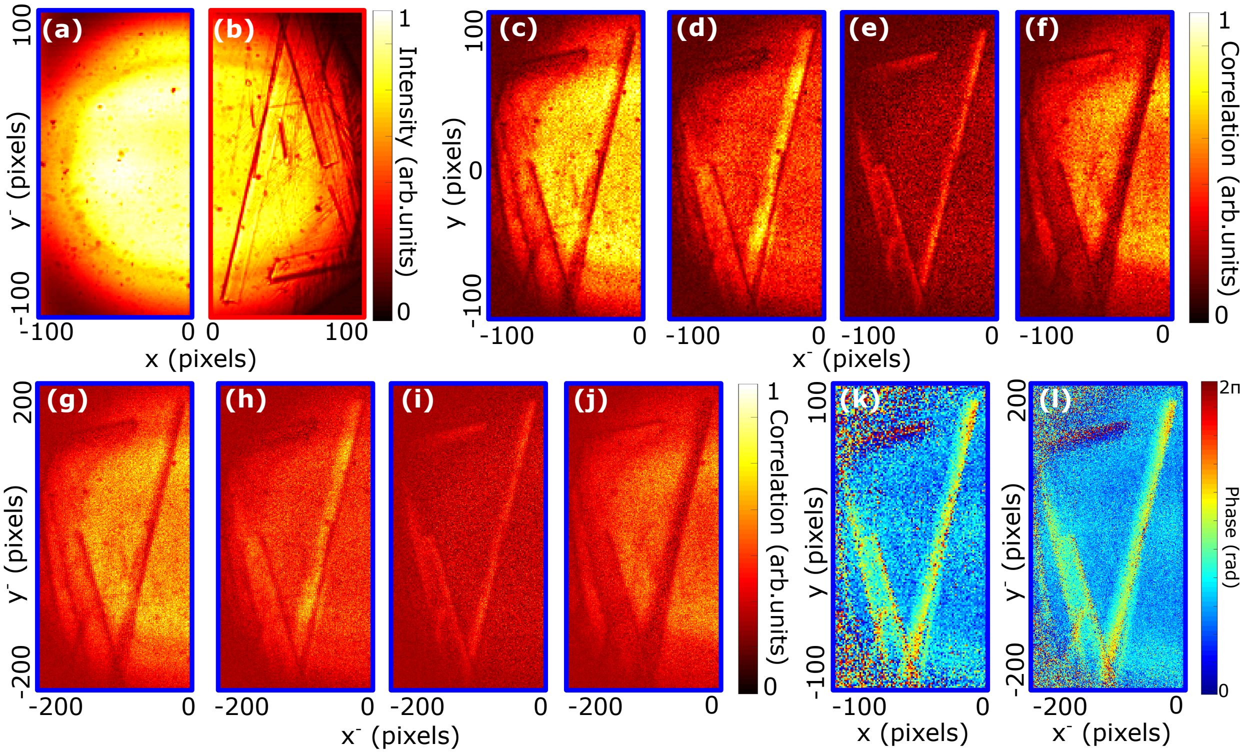

Supplementary Figure 9: ***Pixel super-resolution in entanglement-enabled quantum holography.*** ***(a)*** *and* ***(b)****, Intensity images measured on each half of the camera.* ***c-f****, JPD anti-diagonals* $\Gamma_\alpha(\mathbf{r}, -\mathbf{r})$ *measured for* $\alpha = 0$ *(c),* $\alpha = \pi/2$ *(d),* $\alpha = \pi$ *(e) and* $\alpha = 3\pi/2$ *(f).* ***(g-j)****, JPD minus-coordinate projections* $P^-_\alpha(\mathbf{r}^-)$ *measured for* $\alpha = 0$ *(g),* $\alpha = \pi/2$ *(h),* $\alpha = \pi$ *(i) and* $\alpha = 3\pi/2$ *(j).* ***(k)*** *Phase image retrieved using the anti-diagonal images (same as Figure 4.c of the manuscript).* ***(l)*** *Phase image retrieved using the minus-coordinate projections (same as Figure 4.d of the manuscript).*

5.3 Phase image retrieved using JPD minus-coordinate projections $P^{-}(\mathbf{r}^{-})$ (with pixel super-resolution)

To use our pixel super-resolution approach, the minus-coordinate projections (noted P_{α}^{-}) of the JPD is now considered instead of the anti-diagonal. Taking into account that in our experiment (Figure 4.a of the manuscript) the object t_6 and the SLM are positioned on each halves of the optical plane (i.e. $t_6(x < 0, y) = 1$ and $\alpha(x > 0, y) = 0$), the JPD minus-coordinate is calculated from Equation 39 and simplifies as:

$$P^{-}(\mathbf{r}^{-}) = |t_6(\mathbf{r}^{-}/2)|^2 \left[1 + \cos(\Delta\theta(-\mathbf{r}^{-}/2) - \alpha(\mathbf{r}^{-}/2))\right]^2 \text{ , for all } \mathbf{r} = (x > 0, y) \quad (41)$$

In the holographic reconstruction process, four JPD minus-coordinate projections (noted $P_{\alpha}^{-}(\mathbf{r}^{-})$) are measured for four different uniform phase shifts $\alpha(\mathbf{r}) \in \{0, \pi/2, \pi, 3\pi/2\}$ programmed on the SLM. Figures 9.g-j show the four minus-coordinate projections measured at each phase value. The spatial phase $\Delta\theta$ shown in Figure 9.l (and also in Figure 4.c of the manuscript) is finally reconstructed by combining the four JPD minus-coordination projections using the formula: $\arg[P_0^{-}(\mathbf{r}^{-}) - P_{\pi}^{-}(\mathbf{r}^{-}) + i(P_{\pi/2}^{-}(\mathbf{r}^{-}) - P_{3\pi/2}^{-}(\mathbf{r}^{-}))] = \Delta\theta(\mathbf{r}^{-}/2)$. The resulting phase image has a better spatial resolution than the one shown in Figure 9.k measured using the conventional entanglement-enabled quantum holographic process.

6 Details on full-field NOON state quantum holography

The use of optical NOON-state (N=2) for holographic imaging has been demonstrated by Ono et al [15] using a raster-scanning approach. In our work, we extended it to full-field imaging using the experiment described in Figure 10.a. In such a configuration, photon pairs entangled in space and polarisation pass through an SLM and illuminate a birefringent object t_7. In this case, we use a near-field configuration and the object, SLM and EMCCD camera are all positioned in conjugate image planes (see section 6.1 for more details).

In our experiment, t_7 is a checkerboard birefringent phase pattern composed of 9 square areas with different phase values (Fig. 10.b). The SLM is used to perform a phase-shifting holographic process at four phase values $\{0, \pi/4, \pi/2, 3\pi/4\}$. Sum-coordinate projections of four filtered JPDs are measured at each step of the process and then combined to reconstruct the object phase image shown in Fig. 10.c (see section 6.6 for more details). This image has better pixel resolution than the phase image of the same object retrieved using an equivalent classical holographic system, shown in Fig. 10.d (see section 6.5 for more details).

Furthermore, it is worth noting that phase values in the image retrieved using photon pairs are twice those of the object and the classical phase image, as expected for N00N-state holography and also as demonstrated in previous scanning-based imaging approach with N00N states [15,16]. In addition to preserving this sensitivity enhancement, our full-field version of N00N-state holography then also provides a better pixel resolution, an advantage that could only be matched with the

scanning version by increasing the acquisition time by at least a factor of four. The range of applications can be extended to non-birefringent phase objects by using birefringent plates, as done in differential interference contrast microscopy [17,18].

6.1 Details about the experimental setup

Laser, camera, SLM, filters and photon-pairs source are the same than in Figure 4.a of the manuscript. The two-lens imaging system $f_{14} - f_{15}$ is composed of a series of 4 lenses with focal lengths 45 mm - 75 mm - 50 mm - 150 mm. They are arranged so that the first and last lenses are positioned at focal length from the crystal and the SLM, respectively, and the distance between each pair of lenses equals the sum of their focal lengths. The two-lens imaging system $f_{16} - f_{17}$ is composed of two lenses with focal lengths 100 mm and 150 mm. Similarly, the second two-lens imaging system $f_{18} - f_{19}$ in Figure 10.a is in reality composed of a series of 4 lenses with focal lengths 150mm - 50 mm - 75 mm - 100 mm, arranged as for $f_{14} - f_{15}$. The imaging system magnification 3.3. Photons correlation width in the camera plane is estimated to $\sigma \approx 13\mu$m.

6.2 Theory of full-field NOON state holography

Assuming that photon-pairs are near-perfectly correlated in space [6], the vectorial two-photon wavefunction $\mathbf{\Psi}$ in the object plane of the experiment described in Figure 10.a can be written as:

$$\mathbf{\Psi}(\mathbf{r_1},\mathbf{r_2}) = \delta(\mathbf{r_1}-\mathbf{r_2})\left[t_7^H(\mathbf{r_1})t_7^H(\mathbf{r_2})\mathbf{e_H} + e^{i(\alpha(\mathbf{r_1})+\alpha(\mathbf{r_2}))}t_7^V(\mathbf{r_1})t_7^V(\mathbf{r_2})\mathbf{e_V}\right] \tag{42}$$

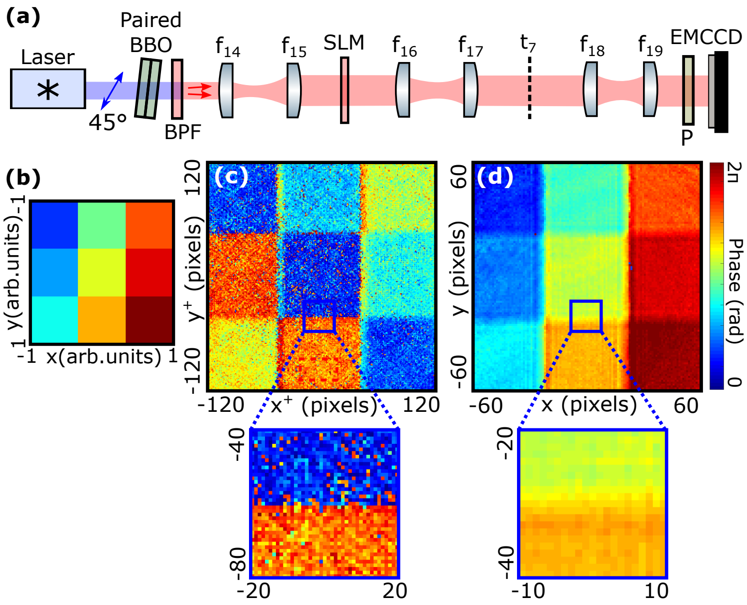

Supplementary Figure 10: ***Results in full-field N00N-state quantum holography.*** ***(a)*** *Experimental setup. Pairs of photons entangled in space and polarization are produced by type I SPDC using the same source of Figure 3.a of the manuscript. Three consecutive two-lens systems* $f_{14} - f_{15}$*,* $f_{17} - f_{18}$ *and* $f_{19} - f_{20}$ *are used to successively image the crystal surface onto a SLM, an object* t_7 *and onto an EMCCD camera. A polarizer (P) at 45°is positioned before the camera. Photons correlation width in the camera plane is estimated to* $\sigma \approx 13\mu m$*. EMCCD camera pixel pitch is* $16\mu m$*.* ***(b)*** *The object* t_7 *is a checkerboard birefringent phase pattern composed of 9 squared areas with different phase values uniformly distributed in* $[0, 2\pi]$ *created using another SLM.* ***(c)*** *Spatial phase image obtained by combining four sum-coordinate projections* P^+ *measured for four different phase shifts* $\{0, \pi/4, \pi/2, 3\pi/4\}$ *programmed by the SLM.* ***(d)*** *Spatial phase of the same object reconstructed by classical holography after replacing the source of photon pairs by a laser at* 810 *nm and* 45° *polarisation and measuring four intensity images measured for four different phase shifts* $\{0, \pi/2, \pi, 3\pi/2\}$*.* 2.10^7 *frames were acquired to retrieve the JPD in each case in approximately* 30 *hours of acquisition.*

where t_7^H (resp. t_7^V) is the horizontal (resp. vertical) polarization component of the polarisation sensitive object t_7, $\mathbf{e_H}$ and $\mathbf{e_V}$ are the unit horizontal and vertical polarisation vectors, δ is the Dirac delta function and $\alpha(\mathbf{r})$ is the phase programmed at position $\mathbf{r}$ of the SLM. In the results shown in Figure 10, t_7 is a birefringent phase object produced by using a second SLM i.e. $t_7^H = e^{\theta_H}$ and $t_7^V = e^{\theta_V}$, where θ_H and θ_V are the horizontal and vertical polarization components of the phase. As a result, after passing through the polariser at $45°$, the JPD measured by the camera takes the form of :

$$\Gamma(\mathbf{r_1}, \mathbf{r_2}) = \delta(\mathbf{r_1} - \mathbf{r_2}) \left[1 + \cos\left(\Delta\theta(\mathbf{r_1}) + \Delta\theta(\mathbf{r_2}) - \alpha(\mathbf{r_1}) - \alpha(\mathbf{r_2})\right)\right]^2 \tag{43}$$

where $\Delta\theta(\mathbf{r}) = \theta_H(\mathbf{r}) - \theta_V(\mathbf{r})$ is the object spatial phase that we are trying to retrieve.

6.3 Double-phase effect in NOON-state holography (N=2).

In the experimental configuration shown in Figure 10.a, photon-pairs accumulate twice the phase of the object when passing through it at a given spatial position. In practice, this double-phase effect is precisely what provides this protocol a better phase sensitivity compared to classical holography, as already demonstrated in previous works using raster-scanning configurations [15,16]. A simple way to visualise it is to measure JPD minus-coordinate projections (noted P_α^-) for different uniform phase pattern α programmed on the SLM with no object in the setup. In this case, P_α^- can be calculated from Equation 43 and takes the following theoretical form:

$$P^-(\mathbf{r}^-) = \left|1 + \cos\left(2\alpha\right)\right|^2 \delta(\mathbf{r}^-) \tag{44}$$

where the double-phase effect is captured by the presence of the factor of 2 next to the SLM phase coefficient α. Figures 11.a-c show JPD minus-coordinate projections measured for different values of phases (a) $\alpha = 0$, (b) $\alpha = \pi/4$ and (c) $\alpha = \pi/2$ using the experimental setup in Figure 10.a with no object. As predicted by Equation 44, correlation value at the center peak (i.e. peak at $\mathbf{r}^- = \mathbf{0}$) varies with the value of α. Figure 11.d shows correlation peak values for 29 values of $\alpha \in [0, 2\pi]$ (red curve). This curve confirms the sine shape with a period of $1/2$ predicted in Equation 44. As a comparison, average intensity values are measured using an equivalent classical holography setup (see section 6.6) for $\alpha \in [0, 2\pi]$ are measured (blue curve). This curve shows the classical sine shape curve with a period of 1 (blue curve). Note that the contrast of these sine shape curves are not unity because the horizontal and vertical polarization components of entangled photons and classical light beams were not perfectly balanced in our experiments.

6.4 NOON-state quantum holography using the JPD diagonal $\Gamma(\mathbf{r}, \mathbf{r})$ (no pixel super-resolution).

In the presence of a birefringent phase object t_7 in the experimental setup, the JPD minus-coordinate projection cannot be used to retrieve its phase component $\Delta\theta(\mathbf{r})$. Instead, the JPD diagonal component $\Gamma(\mathbf{r}, \mathbf{r})$ is considered:

$$\Gamma(\mathbf{r}, \mathbf{r}) = |1 + \cos(2\Delta\theta(\mathbf{r}) - 2\alpha(\mathbf{r}))|^2 \tag{45}$$

In the holographic reconstruction process, four JPD diagonals (noted $\Gamma_\alpha(\mathbf{r}, -\mathbf{r})$) are then measured for four different uniform phase shifts $\alpha(\mathbf{r}) \in \{0, \pi/4, \pi/2, 3\pi/4\}$ programmed on the SLM.

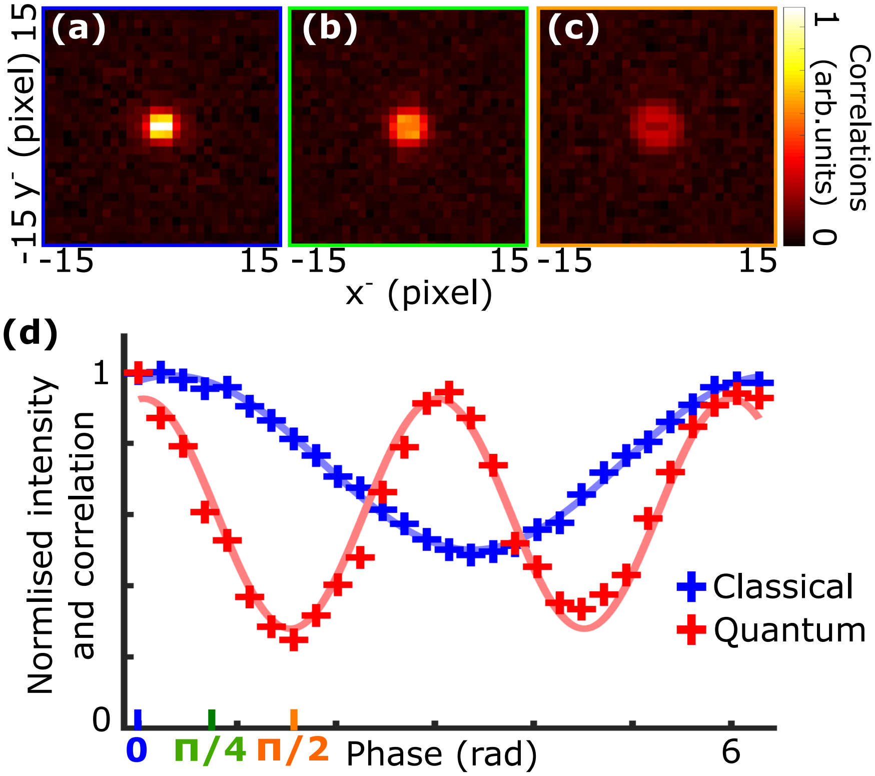

Supplementary Figure 11: ***Double-phase effect.*** ***(a - c)*** *JPD minus-coordinate projections measured using the experiment described in Figure 10.a with no object for different uniform phase values programmed on the SLM (a)* $\alpha = 0$*, (b)* $\alpha = \pi/4$ *and (c)* $\alpha = \pi/2$ *(only central areas of* 30×30 *pixels are shown).* ***(d)*** *JPD minus-coordinate correlation peak values (red curve) and average intensity values measured with an equivalent classical holography setup (blue curve) are represented for different* $\alpha \in [0, 2\pi]$*. The equivalent classical holography setup is described in section 6.6.*

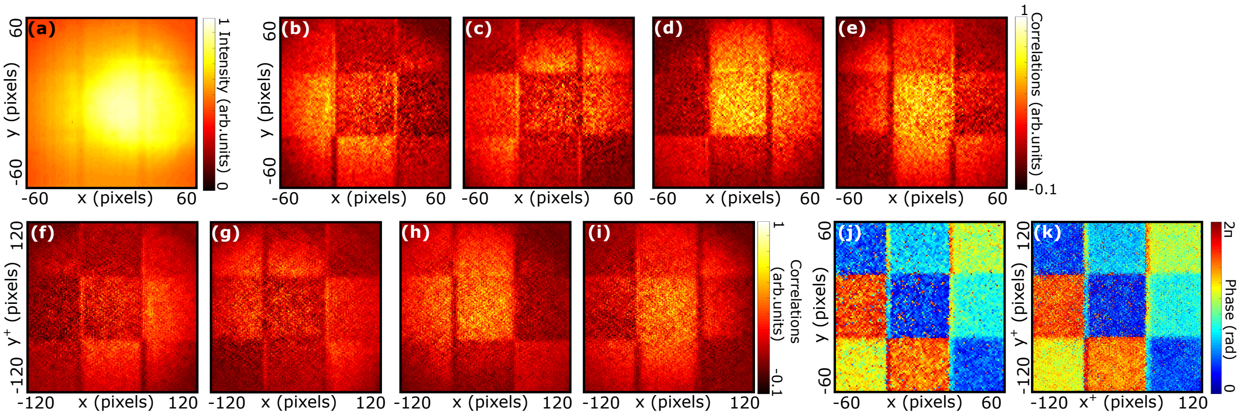

Supplementary Figure 12: ***NOON-state quantum holography using JPD diagonals and sum-coordinate projections.*** ***(a)*** *Constant intensity image acquired by the camera under photon-pairs illumination (not sensitive to the SLM phase).* ***b-e*** *JPD diagonals measured for four different phase values programmed on the SLM (b) $\alpha = 0$, (c) $\alpha = \pi/4$, (d) $\alpha = \pi/2$ and (e) $\alpha = 3\pi/4$.* ***f-i*** *JPD sum-coordinate projections measured for four different phase values programmed on the SLM (f) $\alpha = 0$, (g) $\alpha = \pi/4$, (h) $\alpha = \pi/2$ and (i) $\alpha = 3\pi/4$.* ***(j)*** *Phase image retrieved using the JPD diagonals.* ***(k)*** *Phase image retrieved using the JPD sum-coordinate projections (same as in Figure 10.c).*

Figures 12.b-e show the four diagonal images measured at each phase value. The spatial phase $2\Delta\theta(\mathbf{r})$ shown in Figure 12.j is finally reconstructed by combining these four diagonal images using the formula: $\arg[\Gamma_0(\mathbf{r},\mathbf{r}) - \Gamma_{\pi/2}(\mathbf{r},\mathbf{r}) + i(\Gamma_{\pi/4}(\mathbf{r},\mathbf{r}) - \Gamma_{3\pi/4}(\mathbf{r},\mathbf{r}))] = 2\Delta\theta(\mathbf{r})$. Note that, as predicted by the theory, the reconstructed phase is twice this of the object (i.e. $2\Delta\theta(\mathbf{r})$) because of the double-phase effect.

6.5 NOON-state quantum holography using the JPD sum-coordinate projection P^+ (with pixel super-resolution).

To use our pixel super-resolution approach, the sum-coordinate projections (noted P^+_α) of the JPD are now considered instead of the JPD diagonals. The JPD sum-coordinate projection is calculated using Equation 43:

$$P^+(\mathbf{r}^-) = \left|1 + \cos(\Delta\theta(2\mathbf{r}^+/2) - 2\alpha(\mathbf{r}^+/2))\right|^2 \tag{46}$$

In the holographic reconstruction process, four JPD sum-coordinate projections (noted $P^+_\alpha(\mathbf{r}^-)$) are measured for four different uniform phase shifts $\alpha(\mathbf{r}) \in \{0, \pi/4, \pi/2, 3\pi/4\}$ programmed on the SLM. Figures 12.f-i show the four sum-coordinate projections measured at each phase value. The spatial phase $2\Delta\theta$ shown in Figure 12.l (and also in Figure 10.c) is finally reconstructed by combining these four JPD sum-coordinate projection using the formula: $\arg[P^+_0(\mathbf{r}^+) - P^+_{\pi/2}(\mathbf{r}^+) + i(P^+_{\pi/4}(\mathbf{r}^+) - P^+_{3\pi/4}(\mathbf{r}^+))] = 2\Delta\theta(\mathbf{r}^+/2)$. The resulting phase image has a better spatial resolution than the one shown in Figure 12.k measured using the JPD diagonal. Note that the reconstructed phase is also twice this of the object (i.e. $2\Delta\theta(\mathbf{r})$) because of the double-phase effect.

6.6 Equivalent classical holography setup.

The classical holography setup used to retrieve the phase in Figure 10.d is shown in Figure 13.a. It is the same setup than this described in Figure 10.a after substituting the photon-pairs source by a collimated classical laser beam at 810nm and polarised at $45°$. In the holographic reconstruction

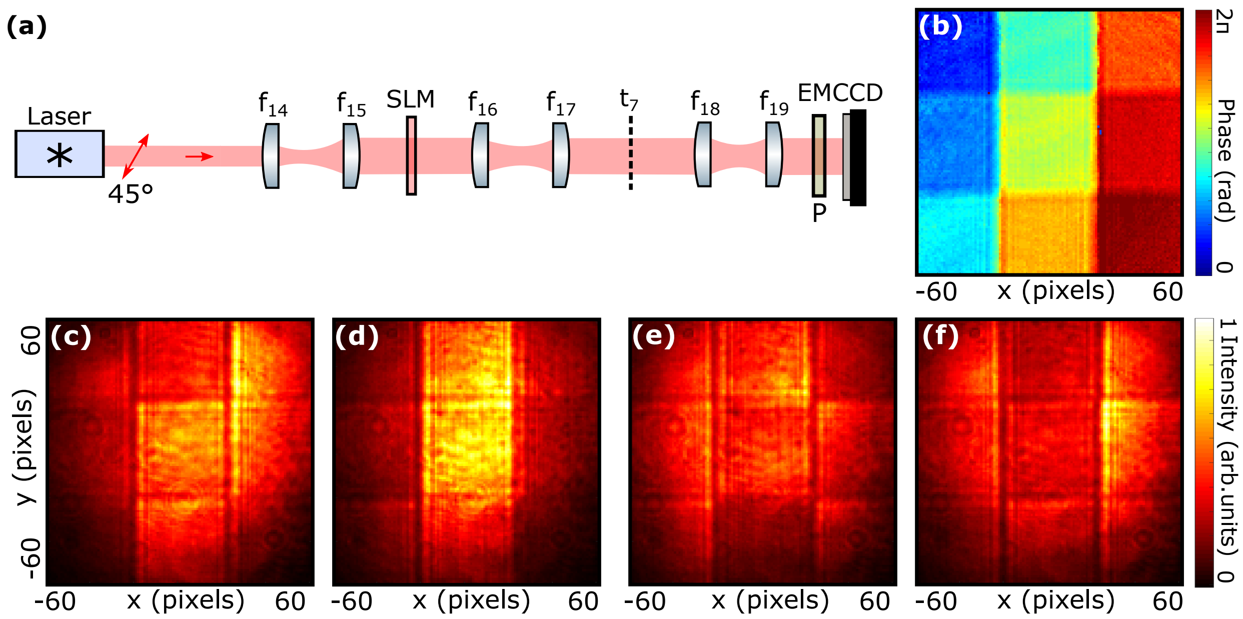

Supplementary Figure 13: ***Equivalent classical holography setup.*** ***(a)*** *Experimental setup used to perform classical holography. It is the same than this described in Figure 10.a after substituting the photon-pairs source by a collimated classical laser beam at* $810nm$ *and polarised at* $45°$*.* ***(b)*** *Phase image retrieved by classical holography (same as Figure 10.d).* ***(c-f)*** *Intensity images measured for four different uniform phase values programmed on the SLM (c)* $\alpha = 0$*, (d)* $\alpha = \pi/2$*, (e)* $\alpha = \pi$ *and (f)* $\alpha = 3\pi/2$*.*

process, four intensity images (noted I^{α}) are measured for four different uniform phase shifts $\alpha(\mathbf{r}) \in \{0, \pi/2, \pi, 3\pi/2\}$ programmed on the SLM. Figures 13.c-f show the four intensity images measured at each phase value. The spatial phase of the object $\Delta\theta$ (Figure 13.b and Figure 10.d) is finally reconstructed by combining these four intensity images using the formula: $\arg[I^0(\mathbf{r}) - I^{\pi}(\mathbf{r}) + i(I^{\pi/2}(\mathbf{r}) - I^{3\pi/2}(\mathbf{r}))] = \Delta\theta(\mathbf{r}/2)$.

6.7 Other artificial and real birefringent phase objects measured using full-field NOON-state holography (N=2).

Figure 14 shows phase images of other artificial and real birefringent objects retrieved by NOON-state quantum holography and classical holography. Note that the range of application of this full-field NOON-state holography technique can be extended to non-birefringent phase objects by using birefringent plates, as done in differential interference contrast microscopy [17].

7 Details on quantum illumination imaging

7.1 First protocol of quantum illumination imaging

The results shown in Figure 3 of the manuscript were obtained using the experimental setup shown in Figure 15.a and the objects shown in Figures 15.b and c.

More details about the experimental setup shown in Figure 15.a: Laser, camera and photon-pairs source are the same than in Figure 1.a. of the manuscript. Exposure time is set to 6 ms. The two-lens imaging system $f_5 - f_6$ is composed of two lenses with focal lengths 35 mm and 75 mm. The single-lens imaging system f_7 is composed of one lens with focal length 50 mm positioned at a distance of 100mm from both objects and the camera. The imaging system magnification 2.1. Photons correlation width in the camera plane is estimated to $\sigma \approx 8\,\mu$m. The same setup was used in [19].

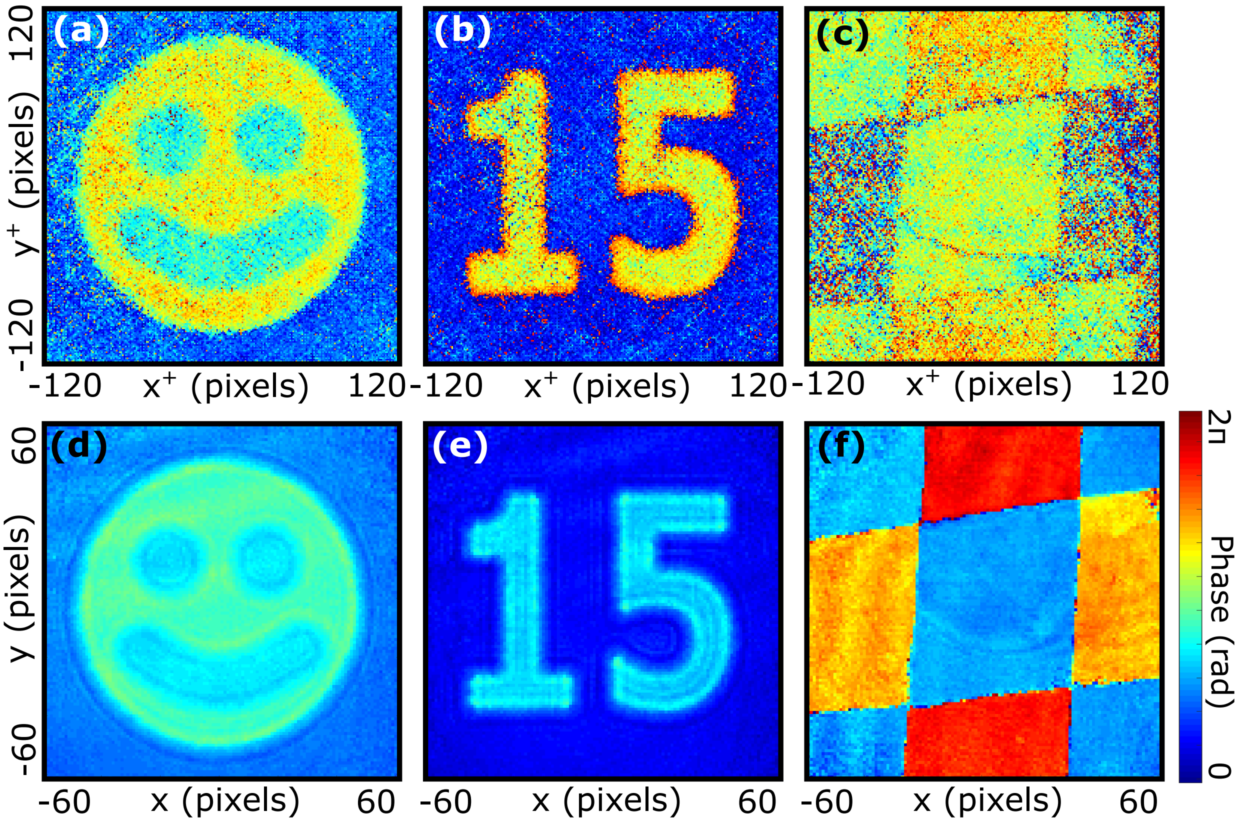

Supplementary Figure 14: ***Phase images of other artificial and real birefringent objects.*** ***(a-c)*** *Phase images of a (a) smiley face and (b) the number 15 created using another SLM retrieved by full-filed NOON-state quantum holography using the sum-coordinate projections. Phase image of (c) pieces of scotch tape forming a cross retrieved by full-filed NOON-state quantum holography (N=2).* ***(d-f)*** *Phase images of the same (d) smiley face, (e) the number 15 and (f) pieces of scotch tape retrieved by classical holography.*

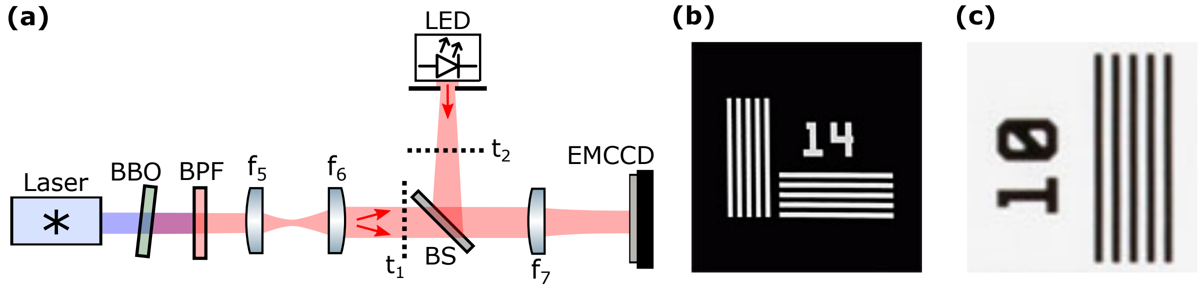

Supplementary Figure 15: ***First protocol of quantum illumination imaging**. **(a)** Experimental setup for quantum illumination in a near-field configuration. Photon pair source is the same as this used in Figure 1.a. of the manuscript. A two-lens system* $f_5 - f_6$ *images the crystal surface onto an object* t_1 ***(b)**, that is in turn imaged onto the EMCCD camera by a single-lens imaging system* f_7. *An object* t_2 ***(c)** is inserted and illuminated by a spatially filtered light-emitting diode (LED) and spectrally filtered at* 810 ± 5 *nm. Images of both objects are superimposed on the camera using a beam splitter (BS).*

7.2 Second protocol of quantum illumination imaging

The second quantum illumination protocol was demonstrated in [3] and its experimental arrangement is shown in Figure 16.a. In such a configuration, an object t_3 (a reflective amplitude object) is illuminated using a far-field configuration and its image is measured with a SPAD camera. Another object t_4 (a transmission object with the same shape as t_3) is illuminated by a classical source and also imaged on the SPAD camera. Objects are positioned so that they overlap exactly on the camera, as shown in the intensity image in Fig. 16.b. In such a far-field configuration, it is important

to understand that information of the JPD is concentrated around the anti-diagonal because photon pairs are spatially anti-correlated in the object plane [5]. Consequently, an image of t_3 can then be retrieved by displaying the main anti-diagonal component $\Gamma_{ij\,-i\,-j}$. Such an anti-diagonal image is the quantity that is conventionally measured and used in all photon-pairs-based imaging schemes using a far-field illumination configuration [3,13,20]. In this case, the JPD pixel super-resolution technique must be adapted by using the minus-coordinate projection P^{-} of the filtered JPD in place of the sum-coordinate projection P^{+} to retrieve the high-resolution image (Fig. 16.d). Even though this image has a better pixel resolution, we also observe that it has a lower signal-to-noise ratio (SNR) and the presence some horizontal dark stripe artefacts. These effects are due to the poor quantum efficiency (2.6%) and fill-factor (10.5%) of the SPAD camera. In addition, note that the object appears twice in the image (the vertical cat-shape and its rotated symmetric copy), which is a known consequence of using a far-field illumination configuration [3,13].

More details about the experimental setup shown in Figure 16.a: BBO crystal has dimensions $5 \times 5 \times 1$mm cut for type-I SPDC at 355nm with a half opening angle of 3 degrees (Newlight Photonics). It is slightly rotated around horizontal axis to ensure near-collinear phase matching of photons at the output (i.e. ring collapsed into a disk). The pump is the third harmonic at 347 nm of a femtosecond pulsed laser with 100 MHz repetition rate, 80 mW average power and beam-diameter of approximately 0.5 mm (Chromacity). The SPAD camera is the *SwissSPAD2* [21]. It has a total of 512×512 pixels, a fill-factor of 10.5%, a photon detection efficiency of 2.6%, a pixel pitch of 16.83 μm and a frame rate of 370 fps. The Fourier-imaging system f_8 is composed of one lens with focal length 45 mm. The two-lens imaging system $f_9 - f_{10}$ is composed of two lenses

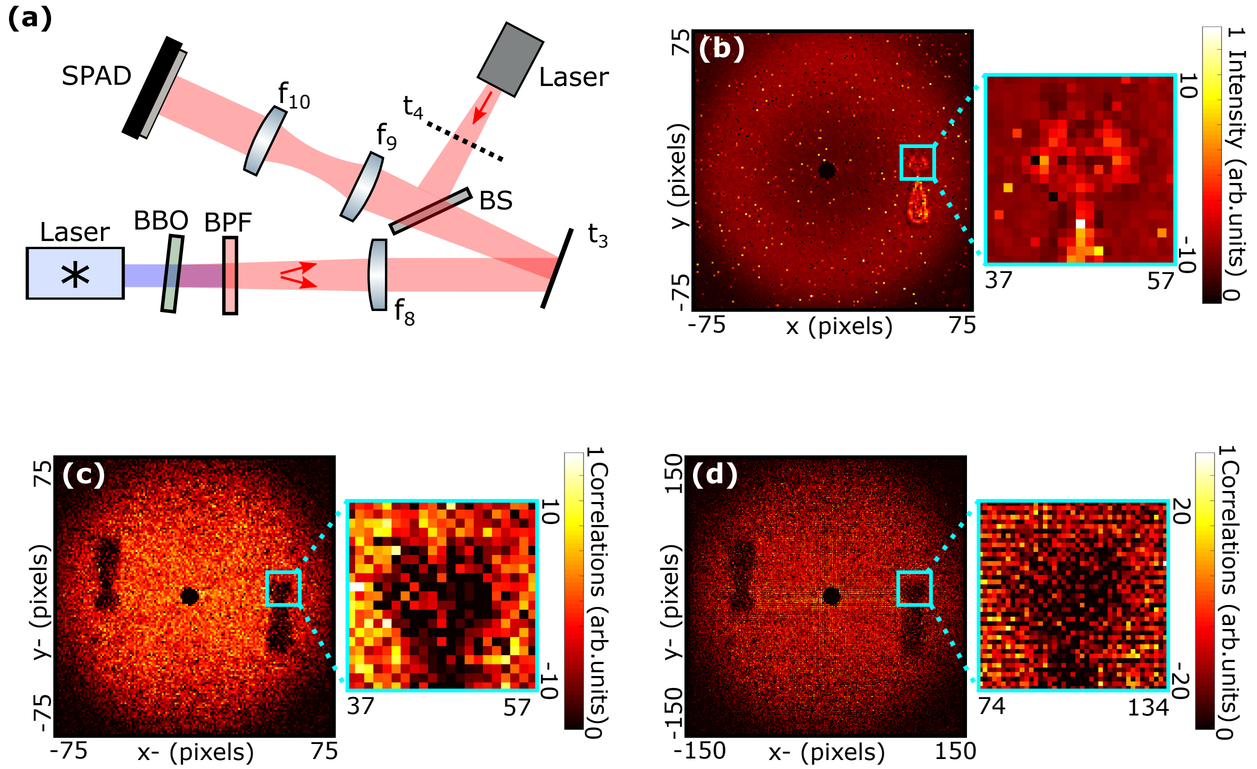

Supplementary Figure 16: ***Results in quantum illumination imaging*****.** ***(a)*** *Experimental setup for quantum illumination in a far-field configuration. Light emitted by a pulsed laser (347 nm) illuminates a BBO crystal (1 mm thickness) to produce spatially entangled photon pairs (694 nm) by type-I SPDC. After the crystal, pump photons are filtered out by a BPF at 694 ± 10 nm. A lens f_8 is used to Fourier-image photon pairs onto a reflective object t_3, that is then imaged onto the SPAD camera by a two-lens system $f_9 - f_{10}$. Another laser (633 nm) illuminates an object t_4 that is also imaged onto the camera using a BS. t_3 is a cat-shape reflective object and t_4 is a transmission object with the same cat-shape, positioned so that the cat shapes overlap on the sensor.* ***(b)*** *Intensity image,* ***(c)*** *anti-diagonal image and* ***(d)*** *minus-coordinate projection of the filtered JPD P^-. Photons correlation width in the camera plane is estimated to $\sigma \approx 8\mu m$. SPAD camera pixel pitch is $16.83\mu m$. 10^7 frames were acquired to retrieve the intensity image and JPD in approximately 7.5 hours of acquisition.*

with focal lengths 100 mm and 50 mm. The imaging system effective focal length is 22.5 mm. Photons correlation width in the camera plane is estimated to $\sigma \approx 8\mu$m.

Supplementary References

1. Defienne, H., Reichert, M. & Fleischer, J. W. General model of photon-pair detection with an image sensor. *Physical review letters* **120**, 203604 (2018).

2. Reichert, M., Defienne, H. & Fleischer, J. W. Massively parallel coincidence counting of high-dimensional entangled states. *Scientific reports* **8**, 7925 (2018).

3. Defienne, H., Zhao, J., Charbon, E. & Faccio, D. Full-field quantum imaging with a single-photon avalanche diode camera. *Physical Review A* **103**, 042608 (2021). URL `https://link.aps.org/doi/10.1103/PhysRevA.103.042608`.

4. Fedorov, M. V., Mikhailova, Y. M. & Volkov, P. A. Gaussian modelling and Schmidt modes of SPDC biphoton states. *Journal of Physics B: Atomic, Molecular and Optical Physics* **42**, 175503 (2009). URL `http://stacks.iop.org/0953-4075/42/i=17/a=175503`.

5. Schneeloch, J. & Howell, J. C. Introduction to the transverse spatial correlations in spontaneous parametric down-conversion through the biphoton birth zone. *Journal of Optics* **18**, 053501 (2016). URL `http://stacks.iop.org/2040-8986/18/i=5/a=053501`.

6. Abouraddy, A. F., Saleh, B. E., Sergienko, A. V. & Teich, M. C. Entangled-photon Fourier optics. *JOSA B* **19**, 1174–1184 (2002). URL `https://www.osapublishing.org/abstract.cfm?uri=josab-19-5-1174`.

7. Chan, K. W., Torres, J. P. & Eberly, J. H. Transverse entanglement migration in Hilbert space. *Physical Review A* **75**, 050101 (2007). URL https://link.aps.org/doi/10.1103/PhysRevA.75.050101.

8. Farsiu, S., Elad, M. & Milanfar, P. A practical approach to superresolution. In *Visual Communications and Image Processing 2006*, vol. 6077, 607703 (International Society for Optics and Photonics, 2006). URL https://www.spiedigitallibrary.org/conference-proceedings-of-spie/6077/60

9. Takeda, H., Farsiu, S. & Milanfar, P. Kernel Regression for Image Processing and Reconstruction. *IEEE Transactions on Image Processing* **16**, 349–366 (2007). Conference Name: IEEE Transactions on Image Processing.

10. Farsiu, S., Robinson, M., Elad, M. & Milanfar, P. Fast and robust multiframe super resolution. *IEEE Transactions on Image Processing* **13**, 1327–1344 (2004). Conference Name: IEEE Transactions on Image Processing.

11. URL https://youtu.be/Zr68ZmksH2U.

12. Burns, P. D. *et al.* Slanted-edge mtf for digital camera and scanner analysis. In *Is and Ts Pics Conference*, 135–138 (SOCIETY FOR IMAGING SCIENCE & TECHNOLOGY, 2000).

13. Defienne, H., Ndagano, B., Lyons, A. & Faccio, D. Polarization entanglement-enabled quantum holography. *Nature Physics* **17**, 591–597 (2021). URL https://doi.org/10.1038/s41567-020-01156-1.

14. Hegazy, S. F. & Obayya, S. S. A. Tunable spatial–spectral phase compensation of type-I (ooe) hyperentangled photons. *JOSA B* **32**, 445–450 (2015). URL https://www.osapublishing.org/josab/abstract.cfm?uri=josab-32-3-445.

15. Ono, T., Okamoto, R. & Takeuchi, S. An entanglement-enhanced microscope. *Nature communications* **4**, 2426 (2013).

16. Israel, Y., Rosen, S. & Silberberg, Y. Supersensitive Polarization Microscopy Using NOON States of Light. *Physical Review Letters* **112**, 103604 (2014). URL https://link.aps.org/doi/10.1103/PhysRevLett.112.103604.

17. Allen, R. D. & David, G. B. The Zeiss-Nomarski differential interference equipment for transmitted-light microscopy. *Zeitschrift fur wissenschaftliche Mikroskopie und mikroskopische Technik* **69**, 193–221 (1969).

18. Terborg, R. A., Pello, J., Mannelli, I., Torres, J. P. & Pruneri, V. Ultrasensitive interferometric on-chip microscopy of transparent objects. *Science Advances* **2**, e1600077 (2016). URL https://advances.sciencemag.org/content/2/6/e1600077.

19. Defienne, H., Reichert, M., Fleischer, J. W. & Faccio, D. Quantum image distillation. *Science Advances* **5**, eaax0307 (2019). URL https://advances.sciencemag.org/content/5/10/eaax0307.

20. Gregory, T., Moreau, P.-A., Toninelli, E. & Padgett, M. J. Imaging through noise with quantum illumination. *Science Advances* **6**, eaay2652 (2020). URL https://advances.sciencemag.org/content/6/6/eaay2652.

21. Ulku, A. C. *et al.* A 512 x 512 SPAD Image Sensor With Integrated Gating for Widefield FLIM. *IEEE Journal of Selected Topics in Quantum Electronics* **25**, 1–12 (2019). Conference Name: IEEE Journal of Selected Topics in Quantum Electronics.
